# Approximate Solution to Nonlinear Dynamics of a Piezoelectric Energy Harvesting Device Subject to Mechanical Impact and Winkler–Pasternak Foundation

**DOI:** 10.3390/ma18071502

**Published:** 2025-03-27

**Authors:** Vasile Marinca, Nicolae Herisanu, Bogdan Marinca

**Affiliations:** 1 Department of Mechanics and Strength of Materials, University Politehnica Timisoara, 300222 Timisoara, Romania; vasile.marinca@upt.ro; 2 Center for Advanced and Fundamental Technical Research, Romanian Academy, 300222 Timisoara, Romania; 3 Department of Applied Electronics, University Politehnica Timisoara, 300006 Timisoara, Romania; bogdan.marinca@upt.ro

**Keywords:** energy harvesting, piezoelectricity, OAFM, Routh–Hurwitz, Lyapunov

## Abstract

To explore the nonlinear dynamics of a piezoelectric energy harvesting device, we consider the simultaneous parametric and external excitations. Based on Bernoulli–Euler beam theory, a new dynamic model is proposed taking into account the curvature of the beam, geometric and electro-mechanical coupling nonlinearities, and damping nonlinearity, with inextensible deformation. The system is discretized by using the Galerkin–Bubnov procedure and then is investigated by the optimal auxiliary functions method. Explicit analytical expressions of the approximate solutions are presented for a complex problem near the primary resonance. The main novelty of our approach relies on the presence of different auxiliary functions, the involvement of a few convergence-control parameters, the construction of the initial and first iteration, and much freedom in selecting the procedure for obtaining the optimal values of the convergence-control parameters. Our procedure proves to be very efficient, simple, easy to implement, and very accurate to solve a complicated nonlinear dynamical system. To study the stability of equilibrium points, the Routh–Hurwitz criterion is adopted. The Hopf and saddle node bifurcations are studied. Global stability is analyzed by the Lyapunov function, La Salle’s invariance principle, and Pontryagin’s principle with respect to the control variables.

## 1. Introduction

Energy has a crucial importance nowadays in the development of society, but unfortunately energy production can generate problems in the environment, since some energy production processes are mainly non-environmentally friendly. For example, the usage of the battery life of wireless sensors or chemical batteries can produce environmental problems or a limited life span. In this context, new methods to convert mechanical energy into electrical energy by means of the piezoelectric effect, electromagnetic induction, or electrostatic induction have been researched. It is known [1,2,3] that piezoelectric energy harvesting (PEH) transforms mechanical vibration into electrical energy. Usually, various technologies are used to convert ambient energy into usable electrical energy. A piezoelectric vibration energy harvester (PEH) is a kind of device that generates maximum power if properly designed. The primary structure of the PEH is a linear resonator as a cantilever beam with a tip mass.

In recent years, all branches of linear or nonlinear vibration energy harvesting are considered to be of fundamental importance to developing cutting-edge technology. Many researchers have intensively investigated mono-stable, bi-stable, or tri-stable nonlinear oscillations with various types of potential wells, considering nonlinear stiffness characteristics. Some of the significant contributions in this field include the analysis of the multiple attractors that may exist across a broad frequency range [1]. Kim et al. [2] derived an analytical expression for the bi- and tri-stable conditions of a multi-stable energy harvester based on bifurcation analyses. It should be emphasized that the triple-well potential has advantageous aspects along the triple-well with a uniform depth. Also, Zhu et al. [3] found that the tri-stable energy harvester exhibits the best performance when the three potential well depths are nearly identical and, in addition, is preferable to the bi-stable energy harvester. Zhou et al. [4] explored the nonlinear effect of energy harvesters by using a mechanical resonator with three potential wells, via multiple solutions, bifurcations, and dynamical hysteresis, pointing out that a good energy harvesting performance is obtained under low frequency and low-level excitations.

Rui et al. [5] examined a rotational energy harvester with limiters to facilitate practical use, showing that stiffness has little influence in limiting effects. Given that the gravity excitation is greater than conventional vibration excitation and large masses are often used in low-frequency applications, large amplitudes pose a significant threat to the life of harvesters. An impact-driving piezoelectric vibration energy harvesting is proposed for low-frequency and broadband vibration based on a layer-separation piezoelectric beam by Cao et al. [6]. A tri-stable piezoelectric energy harvester with two external magnets is analyzed by Wang et al. [7]. Based on the magnetic dipole’s theory, a mechanism for bi- or tri-stability is discussed first, and then a distributed parameter model is explored to study the nonlinear dynamic behaviors and energy generation performance.

Rezaee and Bodaghi [8] explored the instability and nonlinear behaviors of piezoelectric thermal nano-bridges at different temperatures. A computational method called the step-by-step linearization method is then applied to solve the nonlinear equations. The vibration and eigenvalue problem of the actuated nanomanipulator subjected to electrostatic and Casimir attractions are derived. Ma et al. [9] performed the gravitational potential energy of the system, the eccentricity, and rotary inertia of the tip magnet to obtain a more accurate nonlinear three steady-state distribution parameter model of the piezoelectric cantilever energy harvester. The analytic solution of the motion is given by means of multiscale method analysis. Zhang et al. [10] studied the dynamical characteristics of a linear-arch beam tri-stable piezoelectric energy harvester and a magnetic force model by the magnetic dipole method and then used experiments to create a linear-arch composite beam nonlinear restoring force model. The results showed that the system can be mono-stable, bi-stable, or tri-stable by arranging the horizontal or vertical spacing of the magnets under proper excitation.

Chan et al. [11] achieved the mechanical and electrical properties of functionally graded flexo-piezoelectric beams under different electrical boundary conditions. The deflection and induced electric potential are given as analytical expressions for the functionally graded cantilever beam. It was shown that the flexoelectric effect dominates the induced electric potential as the beam thickness decreases. The harmonic balance method is applied to obtain analytical solutions for vertical displacement, output voltage, and power output amplitude by Fang et al. [12]. The difference between the nonlinear lumped-parameter and distributed-parameter models is analyzed, having in view the performance of the energy harvesting system.

Xie et al. [13] used the multiple scale method to obtain an analytic solution of the frequency response curves, and nonlinear characteristics of the energy harvesters are explored under different excitations. A data-driven method to approximate the unknown nonlinear system under the assumption that the nonlinearities can be modeled as continuous functions in a reproducing kernel Hilbert space is presented by Paruchuri et al. [14]. Nonlinearities in a piezoelectric system can arise from internal factors such as nonlinear constitutive laws. In this respect, Song et al. [15] investigated a piezoelectric energy harvester with a double-cantilever beam undergoing coupled bending-torsion vibrations using the width-splitting method in combination with asymmetric mass. The energy harvesting efficiency is evaluated by the proportion of half-power bandwidth and quality factors.

To explore the dynamic behavior of a plucking energy harvester, Noh et al. [16] performed an analytical approach based on the modified differential transform method. The harvester consists of a piezoelectric cantilever oscillator and a rotating plectrum. The effects of plucking speed and overlap length: a series of simulations were carried out. The study of Tion et al. [17] comprises the problem of conventional energy shortages and a non-resonant impact piezoelectric energy capture device by means of piezoelectric film at low frequency. Stress, strain, and output voltage of the piezoelectric film are simulated by COMSOL Multiphysics (https://cn.comsol.com/). The research of Elgamal et al. [18] analyzed challenges of output power with a sharp peak, small bandwidth, and the huge dimensions of the piezoelectric energy harvesters relative to the output power. In this model of a piezoelectric energy harvester array, each beam is made up of three fixed beams that are joined together by a center mass. Zvyagin and Slavin [19] considered piezoelectricity in quantum rare-earth metallic boron oxides with the coupling between magnetic, electric, and elastic subsystems. The change of piezoelectric modules of the considered crystals is proportional to the components of the quadruple susceptibility, which determines the external magnetic and electric fields, strain, and temperature dependence of that change.

Plagianakos et al. [20] showed that the maximization of power harvesting is obtained by prestress calibration and using the model frequency. To analyze and optimize the response of the coupled nonlinear response of the model, a finite element analysis is used. To study the effect of the bending and compression stiffness of a beam, Benhenou et al. [21] proposed a combination of finite element analysis and a dynamic lumped model. They established that low beam compression stiffness can have a negative impact on the performance of the piezoelectric harvester.

Vibration control of a single-beam system by employing nonlinear energy sinks is investigated by Zhao et al. [22]. A numerical procedure is used in the study of the vibration prediction model for the double-beam system with a coupling nonlinear energy sink. The magnetizing current method is examined by Sun et al. [23] to accurately find the system’s magnetic field or magnetic force. A novel penta-stable harvester design is studied by dynamic simulations conducted under Gaussian white noise excitation. Shehuet et al. [24] studied the applications and advancements of piezoelectric energy harvesting in the fields of civil engineering, such as railways, bridges, roadways, buildings, structural health monitoring, and ocean waves. These concerns include issues with integration, limitations in scalability, concerns regarding durability, or inefficient energy conversion.

Yao et al. [25] explored the optimization framework of the cantilevered piezoelectric energy harvester Kirschhoff–Love plate by means of particle swarm optimization in combination with the iso-geometric analysis algorithm to find the optimal design for bridge infrastructure. Based on a MEMS, a novel design strategy is proposed by Nisanth et al. [26] to develop a piezoelectric vibrational energy harvester using the finite element method, parametric analysis, and modeling. The results show that a trapezoidal beam gives a lower resonant frequency than a rectangular beam. Yin et al. [27], on the other hand, proposed a novel rolling-swing electromagnetic energy harvester, amplifying the magnetic field variation within a single cycle of ultra-low-frequency excitation. Experiments proved the potential for powering multiple low-power devices. Singamsetty et al. [28] examined the influence of geometric properties of trapezoidal strain-amplifying sensor/energy harvester on strain amplification with the help of a numerical investigation under a specific set of external loads. This procedure was applied to a real bridge structure.

An analytical model for simulating the nonlinear dynamics of a piezoelectric energy harvesting device is here presented, considering the simultaneous parametric and external excitation. Based on linear Bernoulli–Euler beam theory, accounting for the extended strain given by the curvature of the beam and axial displacement, geometric and electro-mechanical coupling nonlinearities, and damping nonlinearity. Considering the current state of the art, in this paper for the first time we simultaneously consider multiple nonlinearities that appear in the stress, in the curvature, in the strain, or in the Winkler–Pasternak foundation, and in addition, discontinuities described by Heaviside or Dirac functions are considered. In addition, in these conditions, for the first time, the study of local and global stability is developed in the present paper.

The mathematical model is obtained from the extended Hamilton’s principle, and the governing equations are discretized using the Galerkin–Bubnov procedure. The obtained nonlinear differential equations are solved with the help of the optimal auxiliary function method (OAFM).

A very accurate analytical result is provided by the present procedure using a moderate number of convergence-control parameters that are optimally identified using rigorous mathematical approaches. Explicit and very accurate analytical approximate solutions are obtained using only the first iteration, illustrating the real power and effectiveness of the proposed technique.

The local stability of the equilibrium points is studied by means of the homotopy perturbation method and Routh–Hurwitz criteria. The global stability is analyzed by the Lyapunov function, La Salle’s invariance principle, and Pontriagin’s principle with respect to the control variables.

## 2. Derivation of the Governing Equations

**Figure 1:**
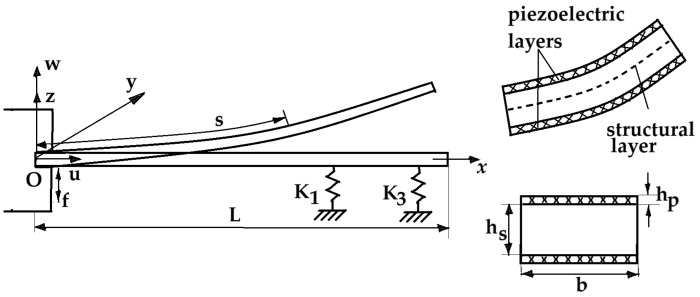
Schematic of the cantilevered piezoelectric beam for an energy harvester.

We consider a uniform bimorph cantilever beam under vertical excitation f(t) resting on a Winkler–Pasternak foundation as depicted in Figure 1. The Winkler–Pasternak foundation model is characterized by a series of close spaces and a shear layer on the springs accounting for the interaction between the springs and the soil.

The beam is modeled as a structural layer and two piezoelectric layers [12,13,14]. A resistance load R_L_ is connected with the piezoelectric layer, which is bounded by two in-plane electrodes assumed to be negligible in thickness compared to the overall thickness of the harvester. Between the piezoelectric layer and the main layer is the gap distance. The base excitation is of the form $f=Asin\bar{\omega }t$ when the driving amplitude A and the frequency ω can vary. The model is excited in close proximity to the fundamental resonance of the coupled electromechanical system. A single-mode approximation is presented, which leads to sufficient accuracy of the nonlinear problem. A smaller stiffness of the piezoelectric layer and the substrate layer harvester can achieve a lower vibration frequency and produce a higher output voltage [6]. The instantaneous electric field induced in the piezoelectric layer is assumed to be uniform throughout the length of the beam. Current and voltage depend either on the electrical load and the piezo harvester, which is commonly modeled as an equivalent current source with a capacitance in parallel.

If L is the length of the beam, h_p_ and h_s_ are the thickness of each piezoelectric layer and the thickness of the substrate layer, respectively, and b is its width, it follows that the height is 2 h_p_ + h_s_. The shearing deformations and rotary motion are supposed to be negligible. In Figure 1, s is the curvilinear coordinate along the middle plane of the beam, and *u*(*s*,*t*) and *w*(*s*,*t*) are the displacements of the beam in the x- and z-axis, respectively. Realistic harvesting circuits, including capacitors or MOSFETs, provide a better estimation of the actual power harvested.

On the basis of Bernoulli–Euler beam theory, the displacement field, including the mid-plane stretching effect along the x, y, and z-axis, can be expressed as follows:

(1) $$\begin{array}{c} U\left(s,t\right)=u\left(s,t\right)-z\frac{\partial w(s,t)}{\partial s}\\ V\left(s,t\right)=0\;\;\;\;\;\;\;\;\;\;\;\;\;\;\;\\ W\left(s,t\right)=w\left(s,t\right)\;\;\;\;\;\;\;\; \end{array}$$

where *U* and *W* are longitudinal and transversal displacements, and ∂w∂s is the rotation angle of the cross-section about the y-axis. The strain of the beam caused by the axial displacement is given by the following:

(2) $$\epsilon_{1}=\sqrt{{\left(1+\frac{\partial u}{\partial s}\right)}^{2}+{\left(\frac{\partial w}{\partial s}\right)}^{2}}-1$$

in which, using the series expansion to the fourth order, the last equation becomes the following:

(3) $$\epsilon_{1}=\frac{\partial u}{\partial s}+\frac{1}{2}{\left(\frac{\partial w}{\partial s}\right)}^{2}-\frac{1}{8}{\left(\frac{\partial w}{\partial s}\right)}^{4}$$

The strain caused by the curvature of the beam to the material point located at a distance z from the middle plane is as follows:

(4) $$\epsilon_{2}=-z\frac{\partial^{2}w}{\partial s^{2}}{\left[1+{\left(\frac{\partial w}{\partial s}\right)}^{2}\right]}^{-3/2}$$

After some manipulations, the strain ϵ can be rewritten as follows:

(5) $$\epsilon =\frac{\partial u}{\partial s}+\frac{1}{2}{\left(\frac{\partial w}{\partial s}\right)}^{2}-\frac{1}{8}{\left(\frac{\partial w}{\partial s}\right)}^{4}-z\left[\frac{\partial^{2}w}{\partial s^{2}}-\frac{3}{2}\frac{\partial^{2}w}{\partial s^{2}}{\left(\frac{\partial w}{\partial s}\right)}^{2}+\frac{15}{8}\frac{\partial^{2}w}{\partial s^{2}}{\left(\frac{\partial w}{\partial s}\right)}^{4}\right]$$

Now, supposing that the strain caused by the axial displacement is negligible (or equivalently, assuming that the beam is inextensible), from Equation (3) it is obtained by the following:

(6) $$u\left(s,t\right)=-\frac{1}{2}\int_{0}^{s}{W^{\prime }}^{2}\left(s,t\right)ds+\frac{1}{8}\int_{0}^{s}{W^{\prime }}^{4}\left(s,t\right)ds$$

where the prime denotes derivatives with respect to the variable s.

The enthalpy density for a linear piezoelectric cantilever will be as follows:

(7) $$H=\frac{1}{2}C_{ijkl}^{c}S_{ij}S_{kl}-e_{mij}-\frac{1}{2}E_{im}^{c}E_{i}E_{m}$$

where *C_ijkl_*, *S_ij_*, *e_mij_*, *E_m_*, and $E_{im}^{c}$ are the Young’s modulus, strain, piezoelectric coupling, electric field, and permittivity tensors, respectively. The superscript *c* means that these constants are measured when the electric field and strain are held constant. Equation (7) is written using the Einstein summation convention.

According to thermodynamics, the stress and electric displacement are defined as follows:

(8) $$T_{ij}={\left.\frac{\partial H}{\partial S_{ij}}\right|}_{s,c};\;D_{i}=-{\left.\frac{\partial H}{\partial E_{i}}\right|}_{s,c}$$

For the particular case of the piezoelectric bimorph beam, the linear constitutive equation can be expressed as follows [13]:

(9) $$\left(\begin{array}{c} T_{1}^{s}\\ T_{1}^{p}\\ D_{3} \end{array}\right)=\left(\begin{array}{ccc} Y_{s} & 0 & 0\\ 0 & Y_{p} & -e_{31}\\ 0 & e_{31} & \epsilon_{33}^{c} \end{array}\right)\left(\begin{array}{c} S_{1}^{s}\\ S_{1}^{p}\\ E_{3} \end{array}\right)$$

where *T*_1_ and *S*_1_ are the stress and strain, respectively; *D*_3_ is the electric displacement; *Y* is Young’s modulus; *e*_31_ = *d*_31_*Y_p_*; *d*_31_ is the piezoelectric strain coefficient; *ε*_33_ is the permittivity at constant strain; and subscript/superscript p and s represent the piezoelectric layers and substrate layer, 1 and 3 indicate the x- and z-direction, and *E*_3_ is the electric field:

(10) $$E_{3}=-\frac{V(t)}{2h_{p}}$$

*V*(*t*) is the electric voltage.

The nonlinear governing equations of the cantilevered piezoelectric beam are derived from the extended Hamilton’s principle by variational identity:

(11) $$\delta \int_{t_{1}}^{t_{2}}\left(T-U+D+W\right)dt=0$$

with the kinetic energy *T*, potential energy *U*, the work performed by the damping force *D*, and electrical energy *W*. The kinetic energy of the beam is as follows:

(12) $$T=\frac{1}{2}\int_{0}^{L}m\left[{\left(\frac{du(s,t)}{dt}\right)}^{2}+{\left(\frac{\partial w}{\partial t}+\frac{\partial f}{\partial t}\right)}^{2}\right]$$

where *m* = 2*ρ_p_bh_p_* + *ρ_s_h_s_*, *ρ_p_*, and *ρ_s_* are the density of piezoelectric layers and substrate layer, respectively.

The potential energy *U* of the substrate and piezoelectric layer is as follows:

(13) $$U=\frac{1}{2}\int_{V_{p}}T_{1}^{p}S_{1}^{p}dV_{p}+\frac{1}{2}\int_{V_{s}}T_{1}^{s}S_{1}^{s}dV_{s}$$

where *V_p_* and *V_s_* are the volumes of two piezoelectric layers and of one substrate layer. Considering the geometric nonlinearity, the strain $S_{1}^{p}$ is written as follows:

(14) $$S_{1}^{p}=S_{1}^{s}=-zw”\left(1-\frac{3}{2}{w^{\prime }}^{2}+\frac{15}{8}{w^{\prime }}^{4}\right)$$

By means of Equations (9) and (14), the potential (13) can be rewritten in the form:

(15) $$\begin{array}{c} U=-\frac{1}{2}\int_{0}^{L}\{Y_{1}bd_{31}R_{L}\left(h+\frac{1}{2}h_{s}\right)w”\left(1-\frac{3}{2}{w^{\prime }}^{2}+\frac{15}{8}{w^{\prime }}^{4}\right)\frac{dQ}{dt}[H\left(x\right)-H(x\\ -L)]+YI\left[{w”}^{2}{\left(1-\frac{3}{2}{w^{\prime }}^{2}+\frac{15}{8}{w^{\prime }}^{4}\right)}^{2}\right]\}ds \end{array}$$

where

(16) $$YI=Y_{s}I_{s}+\frac{2}{3}Y_{p}b\left[3h^{2}h_{s}+{\left(\frac{h}{2}+h_{s}\right)}^{3}-{\left(\frac{h}{2}\right)}^{3}\right],\;{\;Y}_{s}I_{s}=Y_{s}bh_{s}^{3}/Q,\;h=\frac{1}{2}h_{p}$$

and *H*(∙) is the Heaviside function. The Heaviside function is present to point out that the derivative of the function *Q* is defined only in the domain [0,*L*] and to indicate the impact event.

The variation of the work performed by the damping force and Winkler–Pasternak foundation is as follows:

(17) $$\delta D=-\int_{0}^{L}\left[\left(c_{1}\dot{w}+c_{2}\dot{w}\left|\dot{w}\right|\right)\delta w-R_{L}\frac{dQ}{dt}\delta Q+(K_{1}w+K_{3}w^{3})\delta w\right]ds$$

where *c*_1_ and *c*_2_ are the linear and nonlinear quadratic damping coefficients, respectively, and the dot denotes the derivative with respect to time.

The electrostatic energy stored in the piezoelectric layer is as follows:

(18) $$W=\frac{1}{2}\int_{V_{p}}E_{3}D_{3}dV_{p}$$

which can be rewritten as follows:

(19) $$W=\frac{1}{2}Y_{p}bd_{31}R_{L}\left(h+\frac{1}{2}h_{p}\right)\int_{0}^{L}w”{\left(1-\frac{3}{2}{w^{\prime }}^{2}+\frac{15}{8}{w^{\prime }}^{4}\right)}^{2}\frac{dQ}{dt}ds+bLe_{31}^{c}\frac{R_{L}^{2}}{4h_{p}}{\left(\frac{dQ}{dt}\right)}^{2}$$

Now, performing a series of variational manipulations and neglecting the terms of seventh order, the governing equations of the cantilever bimorph piezoelectric beam subject to mechanical impact and nonlinear foundation are obtained as follows:

(20) $$\begin{array}{c} m\ddot{w}\left(s,t\right)+\frac{1}{2}m\left[w^{\prime }\left(s,t\right)\int_{0}^{L}\frac{d^{2}}{dt^{2}}{\left(w^{\prime }\left(s,t\right)\right)}^{2}ds-w”(s,t)\int_{s}^{L}\int_{0}^{s}\frac{d^{2}}{dt^{2}}w”\left(s,t\right)ds\right]+\\ c_{1}\dot{w}\left(s,t\right)+c_{2}\dot{w}\left(s,t\right)\left|\dot{w}\left(s,t\right)\right|+YI\{w^{IV}\left(s,t\right)\left[1-\frac{3}{2}{w^{\prime }}^{2}\left(s,t\right)+\frac{15}{8}{w^{\prime }}^{4}\left(s,t\right)\right]-\\ 9w^{\prime }\left(s,t\right)w”(s,t)w^{\prime \prime \prime }(s,t)-3{w”}^{3}(s,t)+\frac{45}{2}{w^{\prime }}^{2}(s,t)w”(s,t)+\\ \frac{45}{2}{w^{\prime }}^{3}(s,t)w^{\prime \prime \prime }(s,t)\}+K_{1}w+K_{3}w^{3}-aV\left(s,t\right)[1-\frac{3}{2}{w^{\prime }}^{2}\left(s,t\right)+\\ \frac{15}{8}{w^{\prime }}^{4}\left(s,t\right)]\left[\delta \left(s\right)-\delta \left(s-L\right)\right]-3w”(s,t)w^{\prime }(s,t)\left[1-\frac{5}{4}{w^{\prime }}^{2}\left(s,t\right)\right][\delta \left(s\right)-\\ \delta \left(s-L\right)]=-m\ddot{f}(t) \end{array}$$

(21) $$c_{p}\dot{V}\left(s,t\right)+\frac{V(s,t)}{R_{L}}+a\int_{0}^{L}\frac{d}{dt}\left[w”(s,t)\left(1-\frac{3}{2}{w^{\prime }}^{2}\left(s,t\right)+\frac{15}{8}{w^{\prime }}^{4}\left(s,t\right)\right)\right]ds=0$$

where $a=Y_{p}bd_{31}\left(h+\frac{3}{2}h_{s}\right),c_{p}=h\varepsilon_{23}^{c}L/(2h_{s})$, and *δ* is the Dirac delta function.

Parametric and external excitation are based on stress, strain, electric displacement, piezoelectric strain displacement, permittivity, velocity, and the piezoelectric layer, linear and nonlinear terms induced by the Winkler–Pasternak foundation, linear and nonlinear damping, and the amplitude of the perturbed force, as presented above.

In order to simplify the governing Equations (20) and (21), the following dimensionless notations are introduced:

(22) $$\begin{array}{c} \bar{s}=\frac{s}{L},\;\bar{w}=\frac{w}{L},\;\bar{f}=\frac{f}{L},\;\bar{c}=\frac{c}{L}\sqrt{\frac{YI}{m}},\;{\bar{c}}_{1}=\frac{c_{1}L^{2}}{\sqrt{YIm}},{\;\bar{c}}_{2}=\frac{c_{2}L}{m},\;\bar{V}=\frac{V}{m}\sqrt{c_{p}YI},\;\mu =\\ \frac{L^{2}}{c_{p}}\sqrt{\frac{m}{YI}},\;\bar{a}=\frac{a}{\sqrt{YIc_{1}}},\;{\bar{K}}_{1}=\frac{K_{1}}{m}\sqrt{c_{p}YI},\;{\bar{K}}_{3}=\frac{K_{1}L^{2}\sqrt{c_{p}YI}}{m} \end{array}$$

Nonlinearities are given by the strain (31), the curvature (4), the potential (15), the Winkler–Pasternak foundation (17), and damping (17). The discontinuities are given by the Heaviside function (15) and the Dirac function (20). In this way, omitting the bar, the governing Equations (21) and (22) become the following:

(23) $$\begin{array}{c} \ddot{w}\left(s,t\right)+w^{\prime }\left(s,t\right)\int_{0}^{s}\frac{d^{2}}{dt^{2}}\left({w^{\prime }}^{2}\left(s,t\right)\right)ds-w\left(s,t\right)\int_{s}^{1}\int_{0}^{s}\left(\frac{d^{2}}{dt^{2}}\left({w^{\prime }}^{2}\left(s,t\right)\right)\mathrm{ds}\right)\mathrm{ds}\\ +c_{1}\dot{w}+c_{2}\dot{w}\left|\dot{w}\right|+w^{\mathrm{IV}}\left(s,t\right)\left(1-\frac{3}{2}{w^{\prime }}^{2}\left(s,t\right)+\frac{15}{8}{w^{\prime }}^{4}\left(s,t\right)\right)-\\ 9w^{\prime }(s,t)w”\left(s,t\right)w^{\prime \prime \prime }\left(s,t\right)-3{w”}^{3}\left(s,t\right)+\frac{45}{2}{w^{\prime }}^{2}(s,t)w”(s,t)[{w”}^{2}\left(s,t\right)+\\ w^{\prime }\left(s,t\right)w^{\prime \prime \prime }(s,t)]+K_{1}w+k_{3}w^{3}-aV\left(1-\frac{3}{2}{w^{\prime }}^{2}\left(s,t\right)+\frac{15}{8}{w^{\prime }}^{4}\left(s,t\right)\right)[\delta^{\prime }\left(s\right)-\\ \delta^{\prime }(s-1)]-3w^{\prime }(s,t)w”(s,t)\left(1-\frac{5}{4}{w^{\prime }}^{2}(s,t)\right)\left[\delta \left(s\right)-\delta (s-1)\right]=f_{0}sin\bar{\omega }t \end{array}$$

(24) $$\begin{array}{c} \dot{V}+\mu V+a\int_{0}^{1}[{\dot{w}}^{\prime \prime }(s,t)-\frac{3}{2}{\dot{w}}^{\prime \prime }(s,t)-3w”\left(s,t\right)w^{\prime }\left(s,t\right){\dot{w}}^{\prime }\left(s,t\right)\\ +\frac{15}{8}{\dot{w}}^{\prime \prime }\left(s,t\right){w^{\prime }}^{4}(s,t)+\frac{15}{2}{\dot{w}}^{\prime \prime }(s,t)w^{\prime }(s,t){\dot{w}}^{\prime }(s,t)]ds=0 \end{array}$$

According to the Galerkin–Bubnov method, the solution of Equations (23) and (24) may be written in the discretized form. It is considered that the solutions of (23) and (24) can be written in the following forms:

(25) $$w\left(s,t\right)=\sum_{i=1}^{n}X_{i}\left(s\right)T_{1i}\left(t\right),\;V\left(s,t\right)=\sum_{i=1}^{n}X_{i}\left(s\right)T_{2i}\left(t\right)$$

in which the eigenvalues of the cantilever are as follows:

(26) $$X_{i}\left(s\right)=cosh\alpha_{i}s-cos\alpha_{i}s+\frac{cos\alpha_{i}+cosh\alpha_{i}}{sin\alpha_{i}+sinh\alpha_{i}}(sin\alpha_{i}s-sinh\alpha_{i}s)$$

where *α_i_* are the eigenfrequencies of the cantilevered beam and can be determined from the frequency equation $cos\alpha_{i}cosh\alpha_{i}+1=0$.

From this last equation, the first four eigenfrequencies are $\alpha_{1}=1.875,\alpha_{2}=4.6941,\alpha_{3}=7.8547,\alpha_{4}=10.9156$

By substituting Equation (26) into Equations (23) and (24), multiplying by *X_i_*(*s*), then integrating along the beam and considering the relations:

(27) $$\frac{d}{ds}H\left(s\right)=\delta \left(s\right);$$

(28) $$\int_{0}^{L}\frac{d^{2}}{ds^{2}}\delta (s-s_{0})g\left(s\right)ds={\left(-1\right)}^{2}\frac{d^{2}g}{ds^{2}}(s_{0})$$

two nonlinear ordinary differential equations of motion are obtained. By taking n = 1 (first mode), these become the following:

(29) $$\begin{array}{c} {\ddot{T}}_{1}+2c_{1}{\dot{T}}_{1}+c_{2}{\dot{T}}_{1}\left|{\dot{T}}_{1}\right|+\omega^{2}T_{1}+a_{2}T_{1}{\ddot{T}}_{1}+a_{3}T_{1}^{3}+a_{4}T_{1}{\dot{T}}_{1}^{2}+a_{5}T_{1}^{5}+\\ T_{2}\left(b_{1}+b_{2}T_{1}^{2}+b_{4}T_{1}^{4}\right)=f_{0}sin\bar{\omega }t \end{array}$$

(30) $${\dot{T}}_{2}+\mu T_{2}+{\dot{T}}_{1}\left(b_{5}+b_{6}T_{1}^{2}+b_{7}T_{1}^{4}\right)=0$$

where the coefficients *a_i_* and *b_i_* are given in the Appendix A.

It is impossible to find the exact solution for the strongly nonlinear differential Equations (29) and (30).

In the following, we will apply OAFM to solve this system to give an analytical approximate solution with high accuracy.

## 3. Basic Ideas of the Optimal Auxiliary Functions Method (OAFM)

Any general nonlinear differential equation can be described by the following equation [29,30,31,32,33]:

(31) $$L\left[T_{i}(t)\right]+N\left[T_{1},T_{2},\ldots ,T_{n},\right]+g_{i}\left(t\right)=0\begin{array}{cc} , & i=1,2,...,n \end{array}\begin{array}{cc} , & t\in D \end{array}$$

with the boundary conditions:

(32) $$B_{i}\left(T_{i}(t),\frac{dT_{i}(t)}{dt}\right)=0\begin{array}{cc} , & i=1,2,...,n \end{array}$$

where *L* and *N* are the linear and nonlinear operators, *g_i_* is a known function, *t* is the independent variable, *T_i_*(*t*) are unknown functions, *B* is the boundary operator, and D is the domain of interest.

The approximate solutions corresponding to Equations (31) and (32) are ${\bar{T}}_{i}(t)$, which contain only two components:

(33) $${\bar{T}}_{i}(t)=T_{i0}(t)+T_{i1}(t,C_{1},C_{2},\ldots C_{p})$$

where *C*_1_, *C*_2_, …, *C_p_* are *p* unknown parameters; *p* is an arbitrary positive integer number. The initial approximation $T_{i0}(t)$ is obtained from the linear differential equation:

(34) $$L\left[T_{i0}(t)\right]+g_{i}\left(t\right)=0,\;{\;B}_{i}\left(T_{i0}(t),\frac{dT_{i0}(t)}{dt}\right)=0\begin{array}{cc} ,\;\; & i=1,2,...,n \end{array}$$

Theoretically, the first approximation $T_{i1}(t,C_{1},C_{2},\ldots C_{p})$ is obtained from the equation:

(35) $$L\left[T_{i1}(t,C_{1},C_{2},\ldots C_{p})\right]+N\left[T_{i0}(t)+T_{i1}(t,C_{1},C_{2},\ldots C_{p})\right]=0,\;\;B\left(T_{i1},\frac{dT_{i1}}{dt}\right)=0$$

Now, it is very important to mention that to cancel the difficulties that arise in solving Equation (35) and to accelerate the convergence of the first approximate solution $T_{i1}(t,C_{1},C_{2},\ldots C_{p})$, we make the following remarks.

In general, the solution of the linear differential Equation (34) can be expressed as follows:

(36) $$T_{i0}(t)=\sum_{j=1}^{m_{1}}K_{j}f_{j}(t)$$

where the coefficients *K_j_*, the functions *f_j_*, and the positive integer m_1_ are known by elementary calculations. On the other hand, the nonlinear operator *N* from Equation (31), calculated for $T_{i0}(t)$ given by Equation (36) may be written in the form:

(37) $$N\left[T_{i0}(t)\right]=\sum_{s=1}^{m_{2}}l_{s}h_{s}(t)$$

in which the coefficients l_s_, the functions *h_s_*(*t*), and the positive integer *m*_2_ are known, and these depend on the initial approximation $T_{i0}(t)$ and evidently on the expression of the nonlinear operator *N*.

In what follows, we should not solve Equation (35); instead, from the theory of differential equations [34], the Cauchy method, the operator method, the method of influence functions, and so on, more conveniently, one considers the first approximation $T_{i1}(t,C_{1},C_{2},\ldots C_{p})$ depending on $T_{i0}(t)$ and $N\left[T_{i0}(t)\right].$ More precisely, $T_{i1}(t,C_{1},C_{2},\ldots C_{p})$ can be determined from the linear equation:

(38) $$\begin{array}{c} L\left[T_{i1}\left(t,C_{1},C_{2},\ldots C_{p}\right)\right]+\sum_{r=1}^{p}C_{r}F_{r}(f_{j}h_{s})=0,\;\;\\ B\left(T_{i1},\frac{dT_{i1}}{dt}\right)=0,\;j=1,2,\ldots ,m_{1};s=1,2,\ldots ,m_{2} \end{array}$$

where, as a novelty, *C_r_* are *p* unknown parameters and *F_r_* are so-called auxiliary functions depending on the functions *f_i_* and *h_s_* involving Equations (36) and (37), respectively. These functions, *f_j_* and *h_s_*, are the sources for the auxiliary functions *F_r_*. It needs to be emphasized that there is a great freedom to choose the values of the positive integer *p* and of the auxiliary functions *F_r_*.

The initial/boundary conditions could be fulfilled by Equation (38) so that finally, Equation (33) responds to all initial/boundary conditions given by Equation (32). In conclusion, the first approximations $T_{i1}(t,C_{1},C_{2},\ldots C_{p})$ are determined from Equation (38), and therefore the approximate solutions of Equations (31) and (32) can be determined from Equations (33), (34), and (38). The unknown parameters C_1_, C_2_,…, Cp can be optimally identified via rigorous mathematical procedures such as the Ritz method, Galerkin method, the least squares method, the collocation method, the Kantorovich method, or by minimizing the square residual error and so on.

In this way, the optimal values of the convergence-control parameters C_1_, C_2_,…,C_1_, and the auxiliary functions F_r_ are known such that the approximate solutions $T_{i}(t)$, I = 1, 2, …, n are well-determined. The accuracy of the results obtained by our proper procedure is growing along with increasing the number p of the convergence-control parameters C_1_, C_2_, …, C_p_ [29].

We should remark that the nonlinear differential Equations (31) and (32) are reduced to only two linear equations, which do not depend on all terms of the nonlinear operator $N\left[T_{i0}(t)\right]$. Our technique led to a very accurate result, is effective, explicit, and provides a rigorous way to control the convergence using only two linear equations in the absence of small or large parameters in the governing equations or in the initial/boundary conditions.

## 4. OAFM for the Cantilever Bimorph Piezoelectric Beam

In the present study, we consider the nonlinear problem near the primary resonance.

The linear and nonlinear operators associated to Equations (29) and (30) are, respectively:

(39) $$\begin{array}{c} L\left[T_{1}\left(t\right)\right]={\ddot{T}}_{1}+\omega^{2}T_{1};\;\\ N\left[T_{1}(t)\right]=2c_{1}{\dot{T}}_{1}+c_{2}{\dot{T}}_{1}\left|{\dot{T}}_{1}\right|+a_{2}T_{1}{\ddot{T}}_{1}+a_{3}T_{1}^{3}+a_{4}T_{1}{\dot{T}}_{1}^{2}+a_{5}T_{1}^{5}\\ +T_{2}\left(b_{1}+b_{2}T_{1}^{2}+b_{4}T_{1}^{4}\right)-f_{0}sin\bar{\omega }t\; \end{array}$$

(40) $$\begin{array}{c} L\left[T_{2}\left(t\right)\right]={\dot{T}}_{2}+\alpha T_{2};\;\\ N\left[T_{2}(t)\right]=(\mu -\alpha )T_{2}+{\dot{T}}_{1}\left(b_{5}+b_{6}T_{1}^{2}+b_{7}T_{1}^{4}\right) \end{array}$$

According to Equation (33), the approximate solution of Equations (29) and (30) can be written as follows:

(41) $$\begin{array}{c} {\bar{T}}_{1}=T_{10}\left(t\right)+T_{11}(t,C_{1},C_{2},\ldots C_{6})\\ {\bar{T}}_{2}=T_{20}\left(t\right)+T_{21}(t,C_{7},C_{8},\ldots C_{12}) \end{array}$$

The initial conditions for Equations (20) and (30) are as follows:

(42) $$T_{1}\left(0\right)=A,\;{\dot{T}}_{1}\left(0\right)=0,\;T_{2}\left(0\right)=B$$

The initial approximation $T_{10}\left(t\right)$ from Equation (41) is obtained as the solution of the linear equation:

(43) $$L\left[T_{10}(t)\right]=0,\;T_{10}\left(0\right)=A,\;{\dot{T}}_{10}\left(0\right)=0$$

which can be rewritten in the form:

(44) $${\ddot{T}}_{10}+\omega^{2}T_{10}=0,\;T_{10}\left(0\right)=A,\;{\dot{T}}_{10}\left(0\right)=0$$

whose solution is as follows:

(45) $$T_{10}\left(t\right)=Acos\omega t$$

Inserting Equation (45) into the second expression of Equation (39), we obtain (A1) from Appendix B. In Equation (A1) we considered the expressions:

(46) $$sign(sin\omega t)=\frac{4}{\pi }(sin\omega t+\frac{1}{3}sin3\omega t+\frac{1}{5}sin5\omega t+\frac{1}{7}sin7\omega t+\ldots )$$

(47) $$\begin{array}{ll} \left|sin\omega t\right|=sin\omega t & \cdot sign\left(sin\omega t\right)\\ & =sin\omega t+\frac{4}{\pi }(sin\omega t+\frac{1}{3}sin3\omega t+\frac{1}{5}sin5\omega t+\frac{1}{7}sin7\omega t\\ & +\ldots ))\; \end{array}$$

(48) $$c_{2}{\dot{T}}_{10}\left|{\dot{T}}_{10}\right|=-\frac{2}{\pi }c_{2}A^{2}\omega^{2}(1-\frac{1}{3}cos2\omega t-\frac{1}{15}cos4\omega t-\frac{1}{35}cos6\omega t+\ldots )$$

and the expression of $T_{20}$, which will be known subsequently.

The first approximation *T*_11_(*t*) from Equation (41) is determined from the linear equation:

(49) $$\begin{array}{c} {\ddot{T}}_{11}+\omega^{2}T_{11}=\alpha_{1}cos3\omega t+\alpha_{2}cos5\omega t+\alpha_{3}cos\bar{\omega }t+\alpha_{4}cos3\bar{\omega }t+\\ \alpha_{5}cos5\bar{\omega }t+\beta_{1}sin\bar{\omega }t+\beta_{2}sin3\omega t+\beta_{3}sin5\omega t+\beta_{4}sin3\omega t+\beta_{5}sin5\omega t \end{array}$$

The solution of Equation (49) is given in Appendix B as (A2).

In order to simplify the expression (A2), the following notations are introduced:

(50) $$\begin{array}{c} C_{1}=A=\frac{\alpha_{3}}{{\bar{\omega }}^{2}-\omega^{2}};\;C_{2}=-\frac{\alpha_{1}}{8\omega^{2}}=\frac{\alpha_{4}}{{9\bar{\omega }}^{2}-\omega^{2}};\;C_{3}=-\frac{\alpha_{2}}{24\omega^{2}}=\frac{\alpha_{5}}{{25\bar{\omega }}^{2}-\omega^{2}};\;C_{4}=\\ \frac{B}{\bar{\omega }}=\frac{\beta_{1}}{{(\bar{\omega }}^{2}-\omega^{2})\omega };\;C_{5}=-\frac{\beta_{2}}{8\omega^{2}}=\frac{\beta_{4}}{{9\bar{\omega }}^{2}-\omega^{2}};\;C_{6}=-\frac{\beta_{3}}{24\omega^{2}}=\frac{\beta_{5}}{{25\bar{\omega }}^{2}-\omega^{2}} \end{array}$$

Substituting notations (50) into Equation (A2), it holds the following:

(51) $$\begin{array}{c} T_{11}(t,C_{1},C_{2},\ldots C_{6})=C_{1}(cos\omega t-cos\bar{\omega }t)+C_{2}(cos3\omega t-cos3\bar{\omega }t)+\\ C_{3}(cos5\omega t-cos5\bar{\omega }t)+C_{4}(\bar{\omega }sin\omega t-\omega sin\bar{\omega }t)+C_{5}(\bar{\omega }sin3\omega t-\omega sin3\bar{\omega }t)+\\ C_{6}\left(\bar{\omega }sin5\omega t-\omega sin5\bar{\omega }t\right) \end{array}$$

The approximate solution of Equations (29) and (43) is obtained from Equations (41), (45), and (51):

(52) $$\begin{array}{c} {\bar{T}}_{1}\left(t,C_{1},C_{2},\ldots C_{6}\right)=Acos\omega t+C_{1}(cos\omega t-cos\bar{\omega }t)+C_{2}(cos3\omega t-cos3\bar{\omega }t)+\\ C_{3}(cos5\omega t-cos5\bar{\omega }t)+C_{4}(\bar{\omega }sin\omega t-\omega sin\bar{\omega }t)+C_{5}(\bar{\omega }sin3\omega t-\omega sin3\bar{\omega }t)+\\ C_{6}\left(\bar{\omega }sin5\omega t-\omega sin5\bar{\omega }t\right) \end{array}$$

As mentioned in Section 3, we have great freedom to choose the auxiliary functions and their number. Also, we can choose other auxiliary functions and other approximate solutions, such as (A3) and (A4) from Appendix B.

The approximate solution of Equation (30) and of the last equation from Equation (43) is as follows:

(53) $${\bar{T}}_{2}\left(t\right)=T_{20}\left(t\right)+T_{21}(t,C_{7},C_{8},\ldots C_{12})$$

The initial approximation $T_{20}\left(t\right)$ can be found from Equation (34), where $g\left(t\right)=-\lambda \Omega e^{-\alpha t}sin\Omega t$, in which *α*, *λ*, and Ω are unknown:

(54) $${\dot{T}}_{20}+\alpha T_{20}=-B\Omega e^{-\alpha t}sin\Omega t\;,\;T_{20}\left(0\right)=B$$

whose solution is as follows:

(55) $$T_{20}(t)=Be^{-\alpha t}cos\Omega t\;$$

The nonlinear operator identified in Equation (40) calculated for $T_{20}(t)$ given by Equation (55) becomes the following:

(56) $$\begin{array}{ll} N\left[T_{20}\right]= & -\left(\alpha -\mu \right)Be^{-\alpha t}cos\Omega t\\ & -A\omega sin\omega t[b_{5}+\frac{1}{2}b_{6}\left(1+cos2\omega t\right)\\ & +\frac{1}{4}b_{7}\left(3+4cos2\omega t+cos4\omega t\right)] \end{array}$$

Taking into consideration the last equation, the first approximation $T_{21}(t,C_{7},C_{8},\ldots C_{12})$ is obtained from the linear differential equation:

(57) $$\begin{array}{ll} {\dot{T}}_{21}+\alpha T_{21}= & e^{-\alpha t}(\gamma_{1}cos\Omega t+\gamma_{2}sin\Omega t+\gamma_{3}cos3\Omega t+\gamma_{4}sin3\Omega t+\gamma_{5}cos5\Omega t\\ & +\gamma_{6}sin5\Omega t+\gamma_{7}cos\Omega t+\gamma_{8}sin3\Omega t+\gamma_{9}cos3\Omega t+\gamma_{10}sin3\Omega t\\ & +\gamma_{11}cos5\Omega t+\gamma_{12}sin5\Omega t+) \end{array}$$

After some simple manipulations, similar to those from *T*_11_, the solution of Equation (57) can be written as follows:

(58) $$\begin{array}{c} T_{21}(t,C_{7},C_{8},\ldots C_{12})=e^{-\alpha t}[C_{7}(cos\Omega t-cos\omega t)+C_{8}(sin\Omega t-sin\omega t)+\\ C_{9}(cos3\Omega t-cos3\omega t)+C_{10}(sin3\Omega t-sin3\omega t)+C_{11}(cos5\Omega t-cos5\omega t)+\\ C_{12}(sin5\Omega t-sin5\omega t)] \end{array}$$

such that the approximate solution of Equations (30) and (43) becomes the following:

(59) $$\begin{array}{c} {\bar{T}}_{2}(t,C_{7},C_{8},\ldots C_{12})=e^{-\alpha t}[Bcos\Omega t+C_{7}(cos\Omega t-cos\omega t)+C_{8}(sin\Omega t-\\ sin\omega t)+C_{9}(cos3\Omega t-cos3\omega t)+C_{10}(sin3\Omega t-sin3\omega t)+C_{11}(cos5\Omega t-\\ cos5\omega t)+C_{12}(sin5\Omega t-sin5\omega t)] \end{array}$$

The optimal values of *C_i_* and *α*, Ω, may be determined by rigorous mathematical approaches: the collocation method, the Galerkin method, the least square residual method, and others.

To prove the accuracy and efficiency of our technique, we consider a particular case that is obtained from the data presented in the Appendix A and the following values:

(60) $$\begin{array}{ll} A=0.08,B= & 0.003,c=0.057,c_{2}=0.19,\omega =0.5,\bar{\omega }=0.51,a_{2}\\ & =1.009,a_{3}=2.493,a_{4}=1.096,a_{5}=0.553,b_{1}\\ & =0.227,b_{2}=1.102,b_{4}=1.211,b_{5}=0.633,b_{6}\\ & =1.055,b_{7}=2.133,f_{0}=0.002,\mu =1.1 \end{array}$$

Applying a collocation approach, the values of the unknown parameters are as follows:

(61) $$\begin{array}{c} \;C_{1}=0.067929232576,C_{2}=-0.0001376412602,C_{3}=-0.000018737655,\\ C_{4}=0.478534206449,C_{5}=-0.002928256204,C_{6}=-0.000597765146,\\ C_{7}=0.091059506233,C_{8}=0.0112658568203,C_{9}=0.000208469402,\\ C_{10}=0.000769084743,\;C_{11}=-0.000104060747,C_{12}=-0.000035912749,\\ \alpha =0.185,\;\Omega =0.39 \end{array}$$

The approximate solutions become the following:

(62) $$\begin{array}{c} {\bar{T}}_{1}\left(t\right)=0.08cos0.5t+0.067929232576(cos0.5t-cos0.51t)-\\ 0.0001376412602(cos1.5t-cos1.53t)-0.000018737655(cos2.5t-\\ cos2.55t)+0.478534206449(0.51sin0.5t-\omega sin0.51t)-\\ 0.002928256204(0.51sin1.5t-0.5sin1.53t)-\\ 0.000597765146\left(0.51sin2.5t-0.5sin2.55t\right) \end{array}$$

(63) $$\begin{array}{c} {\bar{T}}_{2}(t)=e^{-0.185t}[0.003cos0.39t+0.091059506233(cos0.39t-cos\omega 0.5)+\\ 0.0112658568203(sin0.39t-sin0.5t)+0.000208469402(cos1.17t-\\ cos1.5t)+0.000769084743(sin1.17t-sin1.5t)-\\ 0.000104060747(cos1.95t-cos2.5t)-0.000035912749(sin1.95t\\ -sin2.5t)] \end{array}$$

**Figure 2:**
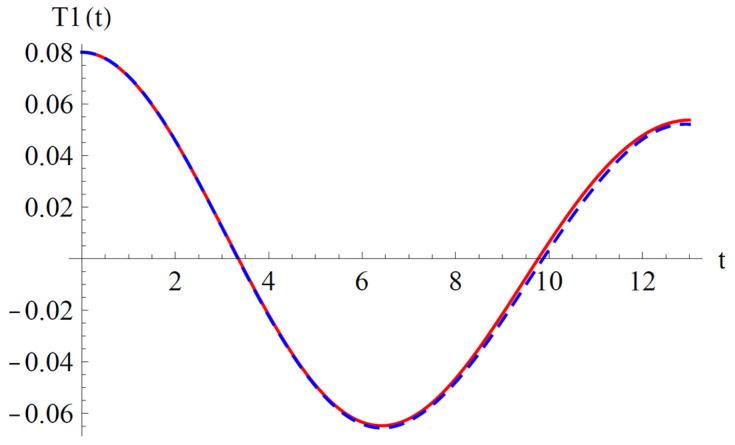
Comparison between the analytical solution (62) and the numerical results for Equations (29) and (43): **_ _ _ _ _** analytical solution (62); **_______ **numerical solution.

**Figure 3:**
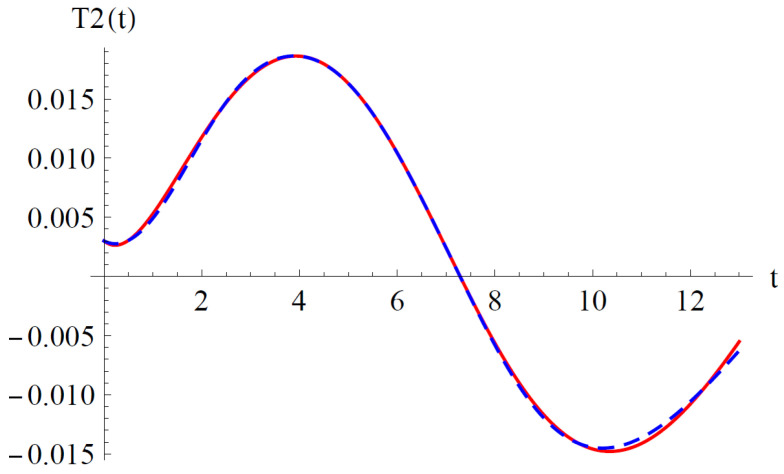
Comparison between the analytical solution (63) and the numerical results for Equations (30) and (43): **_ _ _ _ _** analytical solution (63); **_______ **numerical solution.

To validate the present results derived by OAFM in Figure 2 and Figure 3, a comparison between analytical approximate solutions (62) and (63) and numerical integration results obtained using a classical Runge–Kutta approach is presented.

**Figure 4:**
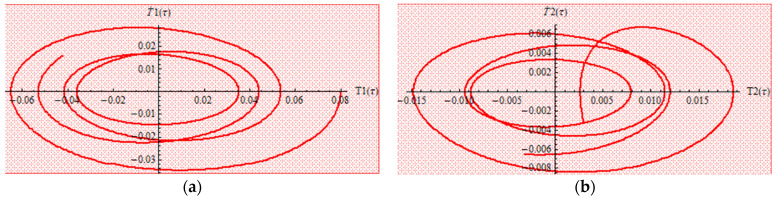
Phase portraits (**a**) in the case of T_1_; (**b**) in the case of T_2_.

One can observe that our approximate analytical solutions of nonlinear differential equations of cantilever bimorph piezoelectric beams, obtained by means of OAFM, are nearly identical to numerical integration results, and this proves the efficiency and high accuracy of our technique. Associated phase plots are presented in Figure 4.

**Figure 5:**
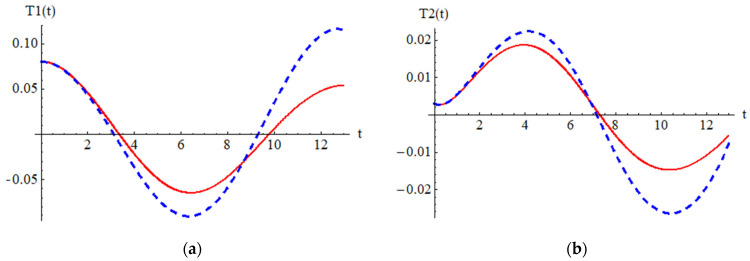
Comparison between damped (red) and undamped (blue) response (**a**) in the case of T_1_; (**b**) in the case of T_2_.

It is important to highlight the effect of damping, which is presented in Figure 5.

**Figure 6:**
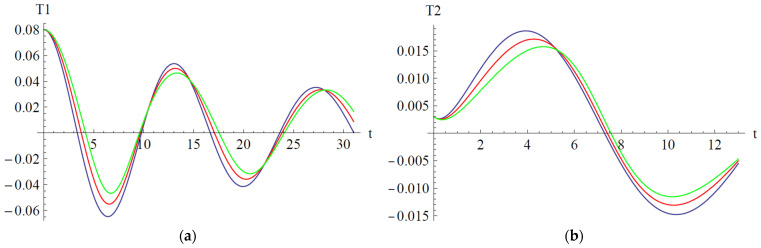
Influence of the parameter a_2_: (**a**) Influence on variable T_1_; (**b**) Influence on variable T_2_; a_2_ = 1.009 (blue), a_2_ = 5.009 (red), a_2_ = 10.009 (green).

**Figure 7:**
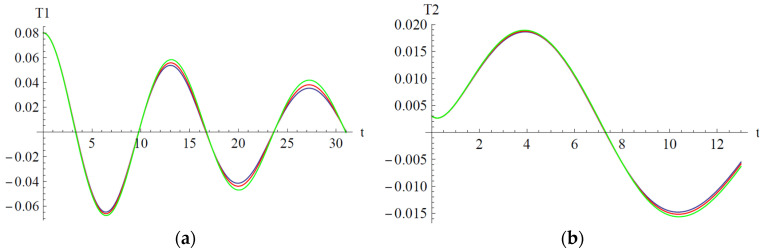
Influence of the parameter b_2_: (**a**) Influence on variable T_1_; (**b**) Influence on variable T_2_; b_2_ = 1.102 (blue), b_2_ = 10.102 (red), b_2_ = 20.102 (green).

**Figure 8:**
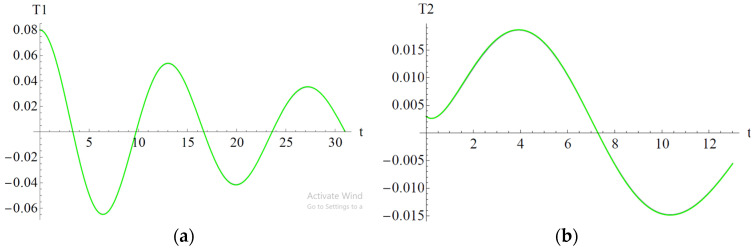
Influence of the parameter b_6_: (**a**) Influence on variable T_1_; (**b**) Influence on variable T_2_; b_6_ = 1.055 (blue), b_6_ = 11.055 (red), b_6_ = 21.055 (green).

In Figure 6, Figure 7 and Figure 8 are depicted the influences of the parameters a_2_, b_2_, and b_6_ on the variables T_1_ and T_2_.

In Figure 6 the influences of the parameter a_2_ are depicted on the variables T_1_ and T_2_. Alternatively, the amplitude of variable T_1_ increases along with increasing the parameter a_2_ on the domain t ∈ [0, 10.5]∪[14, 22] and decreases on the domain t ∈ [10.5, 14]∪[22, 28]. For the variable T_2_, the situation is reversed: alternatively, the amplitude of T_2_ decreases on the domain [0, 5] and increases on the domain [5, 13].

In Figure 7 the influences of parameter b_2_ are depicted on the variables T_1_ and T_2_, respectively. The amplitude of variables T_1_ and T_2_ is variable only in the neighborhood of the extremum points. For both T_1_ and T_2_, the amplitude decreases in the neighborhood of the maximum points and increases in the neighborhood of the minimum point. The situation is more pronounced with the increasing of time.

In Figure 8 the influences of parameter b_6_ are depicted on the variables T_1_ and T_2_. For both variables, the influence of the parameter b_6_ is negligible.

## 5. Stability of the Steady-State Motion of a Piezoelectric Energy Harvesting Device for the Primary Resonance

To study the stability of the steady-state motion near the primary resonance, we propose to distinguish between three time scales by associating a separate independent variable with each one:

(64) $$\xi_{1}=\omega t,\;\xi_{2}=\Omega t,\;\eta =pt$$

We consider the primary resonance for Equation (28): $\omega =\bar{\omega }+\sigma p$, for p = 0, the nonlinear differential equations are reducing to linear equations, and, for p = 1, one finds the original equation, such that Equations (29) and (30) are rewritten as follows:

(65) $$\begin{array}{c} {\ddot{T}}_{1}+\omega^{2}{\dot{T}}_{1}+p[{2c}_{1}{\dot{T}}_{1}+c_{2}{\dot{T}}_{1}\left|{\dot{T}}_{1}\right|+a_{2}T_{1}{\ddot{T}}_{1}+a_{3}T_{1}^{3}+a_{4}T_{1}{\dot{T}}_{1}^{2}+a_{5}T_{1}^{5}+\\ T_{2}\left(b_{1}+b_{2}T_{1}^{2}+b_{4}T_{1}^{4}\right)-f_{0}sin\bar{\omega }t]=0 \end{array}$$

(66) $${\dot{T}}_{2}+\Omega T_{2}+p\left[\mu T_{2}-\Omega T_{2}+{\dot{T}}_{1}\left(b_{5}+b_{6}T_{1}^{2}+b_{7}T_{1}^{4}\right)\right]=0$$

Having in view that Equation (64) is inserted into Equations (65) and (66), we need the following expressions:

(67) $${\dot{T}}_{1}=\frac{dT_{1}}{dt}=\frac{\partial T_{1}}{\partial \xi_{1}}\frac{d\xi_{1}}{dt}+\frac{\partial T_{1}}{\partial \eta }\frac{d\eta }{dt}=\omega \frac{\partial T_{1}}{\partial \xi_{1}}+p\frac{\partial T_{1}}{\partial \eta }$$

(68) $${\ddot{T}}_{1}=\frac{d^{2}T_{1}}{dt^{2}}=\omega^{2}\frac{d^{2}T_{1}}{d\xi_{1}^{2}}+2\omega p\frac{d^{2}T_{1}}{\partial \xi_{1}\partial \eta }+p^{2}\frac{d^{2}T_{1}}{d\eta^{2}}$$

(69) $${\dot{T}}_{2}=\frac{dT_{2}}{dt}=\Omega \frac{\partial T_{2}}{\partial \xi_{2}}+p\frac{\partial T_{2}}{\partial \eta }$$

The variables *T*_1_ and *T*_2_ may be written in a power series of *p*:

(70) $$T_{1}\left(\xi_{1},p\right)=T_{10}\left(\xi_{1},p\right)+pT_{11}\left(\xi_{1},p\right)+p^{2}T_{12}\left(\xi_{1},p\right)+\ldots$$

(71) $$T_{2}\left(\xi_{2},p\right)=T_{20}\left(\xi_{2},p\right)+pT_{21}\left(\xi_{2},p\right)+p^{2}T_{22}\left(\xi_{2},p\right)+\ldots$$

Inserting Equations (67)–(71) into Equations (61) and (66), we obtain the equations from Appendix B.

From these equations, the homotopy orders *p*^0^ and *p*^1^ [35] are, respectively:

(72) $$p^{0}:\;\;\;\;\;\;\;\;\omega^{2}\left(\frac{\partial^{2}T_{10}}{\partial \xi_{1}^{2}}+T_{10}\right)=0$$

(73) $$\;\;\;\;\;\;\;\;\;\Omega^{2}\left(\frac{\partial T_{20}}{\partial \xi_{2}}+T_{20}\right)=0$$

(74) $$\begin{array}{c} p^{1}:\;\;\;\;\;\;\;\;\omega^{2}\left(\frac{\partial^{2}T_{11}}{\partial \xi_{1}^{2}}+T_{11}\right)+2\omega \frac{\partial^{2}T_{10}}{\partial \xi_{1}\partial \eta }+2c_{1}\omega \frac{\partial T_{10}}{\partial \xi_{1}}+c_{2}\omega \frac{\partial T_{10}}{\partial \xi_{1}}\left|\frac{\partial T_{10}}{\partial \xi_{1}}\right|+a_{2}\omega^{2}T_{10}\frac{\partial^{2}T_{10}}{\partial \xi_{1}^{2}}+a_{3}T_{10}^{3}+a_{4}\omega^{2}T_{10}{\left(\frac{\partial T_{10}}{\partial \xi_{1}}\right)}^{2}+a_{5}T_{10}^{5}+\\ b_{1}T_{20}+b_{2}T_{20}T_{10}^{2}+b_{4}T_{20}T_{10}^{4}-f_{0}sin\xi_{1}=0 \end{array}$$

(75) $$\Omega \left(\frac{\partial T_{21}}{\partial \xi_{2}}+T_{21}\right)+\frac{\partial T_{20}}{\partial \eta }+\left(\mu -\Omega \right)T_{20}+b_{5}\omega \frac{\partial T_{10}}{\partial \xi_{1}}+b_{6}\omega T_{10}^{2}\frac{\partial T_{10}}{\partial \xi_{1}}+b_{7}\omega T_{10}^{2}\frac{\partial T_{10}}{\partial \xi_{1}}=0$$

The solutions are, respectively:

(76) $$T_{10}\left(\xi_{1},\eta \right)=A_{1}\left(\eta \right)cos\xi_{1}+A_{2}\left(\eta \right)sin\xi_{1}$$

(77) $$T_{20}\left(\xi_{2},\eta \right)=A_{3}\left(\eta \right)cos\xi_{2}+A_{4}\left(\eta \right)sin\xi_{2}$$

where *A_i_*. *i* = 1, 2, 3, 4 are functions depending only on the variable *η*.

Substituting Equations (76)–(77) into (75) and then avoiding secular terms yields the following:

(78) $$\frac{\partial T_{20}}{\partial \eta }=0$$

from which it is clear that *A*_3_ and *A*_4_ are constant.

From Equation (74), we have the following:

(79) $$2\omega \frac{dA_{1}}{d\tau }-2c_{1}\omega A_{1}+\frac{3}{4}\left(A_{1}^{2}A_{2}+A_{2}^{3}\right)a_{3}+\frac{3}{4}\left(-A_{1}^{2}A_{2}-A_{2}^{3}\right)a_{4}+\left(\frac{5}{8}A_{1}^{4}A_{2}+\frac{5}{8}A_{1}^{2}A_{2}^{3}+\frac{5}{8}A_{2}^{5}\right)a_{5}-f_{0}=0$$

(80) $$-2\omega \frac{dA_{2}}{d\tau }+2c_{1}\omega A_{2}+\frac{3}{4}\left({A_{1}^{3}+A_{1}A}_{2}^{2}\right)a_{3}+\frac{3}{4}\left(A_{1}^{3}-{A_{1}A}_{2}^{2}\right)a_{4}+\left(\frac{5}{8}A_{1}^{5}+\frac{5}{8}A_{1}^{3}A_{2}^{2}+\frac{5}{8}{A_{1}A}_{2}^{4}\right)a_{5}=0$$

The equilibrium points (*A_ie_*, *i* = 1, 2) can be obtained from the condition:

(81) $$\frac{dA_{1e}}{d\tau }=\frac{dA_{2e}}{d\tau }=0$$

The equilibrium points are solutions of the following nonlinear algebraic equations:

(82) $$-2c_{1}\omega A_{1e}+\frac{3}{4}A_{2e}\left(A_{1e}^{2}+A_{2e}^{2}\right)a_{3}+\frac{3}{4}A_{2e}\left(A_{2e}^{2}-A_{1e}^{2}\right)a_{4}+\frac{5}{8}A_{2e}\left(A_{1e}^{4}+A_{1e}^{2}A_{2e}^{2}+A_{2e}^{4}\right)a_{5}-f_{0}=0$$

(83) $$2c_{1}\omega A_{2e}+\frac{3}{4}A_{1e}\left(A_{1e}^{2}+A_{2e}^{2}\right)a_{3}+\frac{3}{4}A_{1e}\left(A_{1e}^{2}-A_{2e}^{2}\right)a_{4}+\frac{5}{8}A_{1e}\left(A_{1e}^{4}+A_{1e}^{2}A_{2e}^{2}+A_{2e}^{4}\right)a_{5}=0$$

To solve these nonlinear equations, we introduce a new parameter ψ:

(84) $$A_{2e}=\psi A_{1e}$$

From Equations (82)–(84) after some manipulations, it can be determined the unknown *ψ* and *A*_1*e*_ are as follows:

(85) $$\begin{array}{c} \frac{\sqrt{9{\left[a_{3}+a_{4}+\left(a_{3}-a_{4}\right)\psi^{2}\right]}^{2}-80a_{5}\omega c_{1}\psi (1+\psi^{2}+\psi^{4})}-3\left[a_{3}+a_{4}+(a_{3}-a_{4})\psi^{2}\right]}{5a_{5}(1+\psi^{2}+\psi^{4})}=\\ {\left[\frac{10f_{0}a_{5}(1+\psi^{2}+\psi^{4})}{20\omega c_{1}a_{5}\left(1+\psi^{2}\right)\left(1+\psi^{2}+\psi^{4}\right)-3a_{4}\psi (1-\psi^{2})\left(\sqrt{9{\left[a_{3}+a_{4}-(a_{3}-a_{4})\psi^{2}\right]}^{2}-3\left(a_{3}+a_{4}+(a_{3}-a_{4})\psi^{2}\right)}\right)}\right]}^{2}\;,\;\psi <0 \end{array}$$

(86) $$A_{1e}=\frac{10f_{0}a_{5}(1+\psi^{2}+\psi^{4})}{20\omega c_{1}a_{5}\left(1+\psi^{2}\right)\left(1+\psi^{2}+\psi^{4}\right)-3a_{4}\psi (1-\psi^{2})\left[\sqrt{9{\left[a_{3}+a_{4}+(a_{3}-a_{4})\psi^{2}\right]}^{2}80a_{5}\omega c_{1}\psi \left(1+\psi^{2}+\psi^{4}\right)-3\left(a_{3}+a_{4}+(a_{3}-a_{4})\psi^{2}\right)}\right]}\;$$

**Figure 9:**
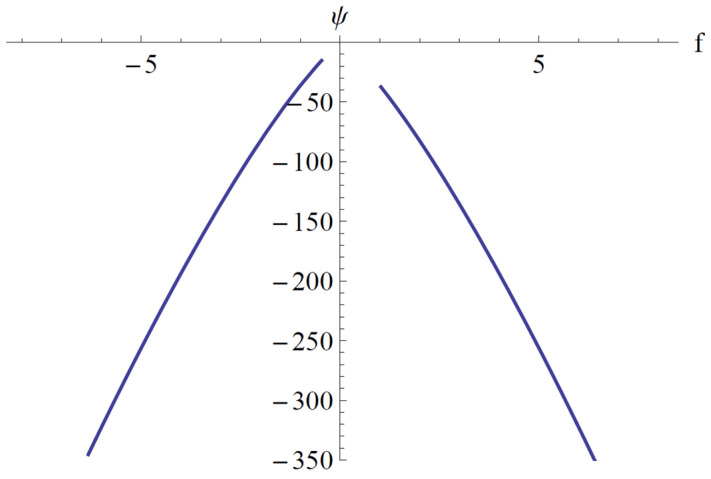
Variation of the parameter ψ with respect to f.

**Figure 10:**
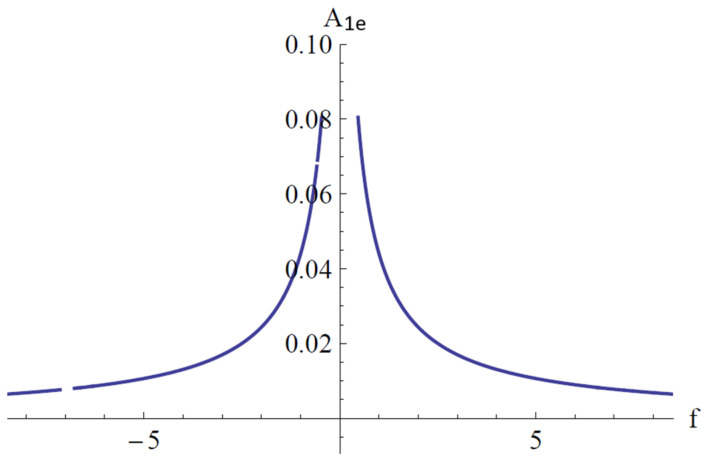
Variation of the parameter A_1e_ with respect to f.

**Figure 11:**
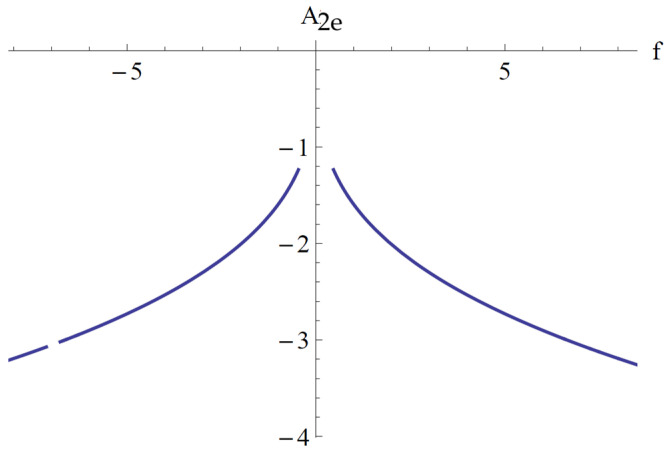
Variation of the parameter A_2e_ with respect to f.

In Figure 9, Figure 10 and Figure 11, the parameter *ψ* given by Equation (85), the points of equilibrium *A*_1*e*_ given by Equation (86), and *A*_2*e*_ given by Equation (84) for the fixed values *a*_2_ = 1.009, *a*_3_ = 2.493, *a*_4_ = 1.096, *a*_5_ = 0.553, *b*_1_ = 0.227, *b*_2_ = 1.102, *b*_4_ = 1.211, and *c*_1_ = 0.057 are depicted.

The stability of steady-state motion is determined using the eigenvalues of the Jacobian matrix derived from Equations (79) and (80):

(87) $$\left[J\right]=\left(\begin{array}{cc} a_{11} & a_{12}\\ a_{21} & a_{22} \end{array}\right)$$

where

(88) $$a_{11}={\left(\frac{\partial A_{1}^{\prime }}{\partial A_{1}}\right)}_{A_{e}};\;a_{12}={\left(\frac{\partial A_{1}^{\prime }}{\partial A_{2}}\right)}_{A_{e}};\;a_{21}={\left(\frac{\partial A_{2}^{\prime }}{\partial A_{1}}\right)}_{A_{e}};\;a_{22}={\left(\frac{\partial A_{2}^{\prime }}{\partial A_{2}}\right)}_{A_{e}},\;A^{\prime }=\frac{dA}{d\eta }$$

The coefficients a_ij_ are as follows:

(89) $$\begin{array}{c} a_{11}=-2\omega c_{1}+\frac{3}{2}a_{3}\psi A_{1e}^{2}+\frac{3}{2}a_{4}\psi A_{1e}^{2}+\frac{5}{4}a_{5}\left(2\psi +\psi^{3}\right)A_{1e}^{4}\\ a_{12}=\frac{3}{4}a_{3}\left(1+3\psi^{2}\right)A_{1e}^{2}+\frac{3}{4}\left(1-3\psi^{2}\right)A_{1e}^{2}a_{4}+\frac{5}{8}\left(1+3\psi^{2}+5\psi^{4}\right)A_{1e}^{4}a_{5}\;\\ a_{21}=\frac{3}{4}a_{3}\left(3+\psi^{2}\right)A_{1e}^{2}+\frac{3}{4}a_{4}\left(3-\psi^{2}\right)A_{1e}^{2}+\frac{5}{8}a_{5}\left(5+3\psi^{2}+\psi^{4}\right)A_{1e}^{4}\;\\ a_{22}=2\omega c_{1}+\frac{3}{2}a_{3}\psi A_{1e}^{2}-\frac{3}{2}a_{4}\psi A_{1e}^{2}+\frac{5}{4}a_{5}\left(\psi +2\psi^{3}\right)A_{1e}^{4}\; \end{array}$$

The signs of the real parts of eigenvalues of the Jacobian matrix are derived from the characteristic equation:

(90) $$det\left(\left[J\right]-\lambda \left[I_{2}\right]\right)=0$$

where [*I*_2_] is the second order unity matrix and *λ* is the eigenvalue of the Jacobian matrix. Considering the expressions (89), the characteristic equation (90) may be written as follows:

(91) $$\lambda^{2}+tr\left[J\right]\lambda +det\left[J\right]=0$$

in which the trace of the Jacobian matrix will be as follows:

(92) $$tr\left[J\right]=-\left(a_{11}+a_{22}\right)=3a_{3}\psi A_{1e}^{2}+\frac{15}{4}a_{5}\left(\psi +\psi^{3}\right)A_{1e}^{4}$$

and the determinant of *J* is as follows:

(93) $$det\left[J\right]=a_{11}a_{22}-a_{12}a_{21}$$

and the discriminant of Equation (91) is as follows:

(94) $$F\left(\psi \right)={\left(tr\left[J\right]\right)}^{2}-4det\left[J\right]$$

The signs of the eigenvalues *λ*_1_ and *λ*_2_ given by the following:

(95) $$\lambda_{1}=-\frac{1}{2}tr\left[J\right]+\frac{1}{2}\sqrt{F\left(\psi \right)},\;\lambda_{2}=-\frac{1}{2}tr\left[J\right]-\frac{1}{2}\sqrt{F\left(\psi \right)}$$

determine local stability. We will leave discussion of so-called “borderline” cases until the end: these borderline cases involve ones in with both eigenvalues:

Case 5.1 Both eigenvalues are real and $F\left(\psi \right)>0$

(a) If $\lambda_{1}<0$ and $\lambda_{2}<0$, this is called a stable nodal point.
(b) If $\lambda_{1}>0$ and $\lambda_{2}>0$, then this is the case of an unstable nodal point.
(c) If $\lambda_{1}>0$ and $\lambda_{2}<0$, this is the case of a saddle point.

Case 5.2 Both eigenvalues are real and $F\left(\psi \right)=0$

(a) $\lambda_{1}=\lambda_{2}<0$, then this case is called a stable nodal point $\lambda_{1}=\lambda_{2}>0$, then this is the case of an unstable node point.

Case 5.3. $det\left[J\right]=0$:

(a) If $\lambda_{1}=0$ and $\lambda_{2}<0$, then this is the case of a stable point but not asymptotically stable.
(b) If $\lambda_{1}=0$ and $\lambda_{2}>0$, then this is the case of an unstable point.
(c) If $\lambda_{1}=\lambda_{2}=0$, this is the case of a stable nodal point if *A*_1*e*_ = *C*_1_ (constant) and *A*_2*e*_ = *C*_2_ (constant).
(d) If $\lambda_{1}=\lambda_{2}=0$, this is the case of an unstable nodal point if $A_{1e}=C_{1}+C_{2}t$, C_i_ = constant, $i=\bar{1,4}$.

Case 5.4. Both eigenvalues are complex: $F\left(\psi \right)<0$ and $\lambda_{1,2}=p\pm qi$, $i^{2}=-1$, q≠0

(a) If *p* < 0 and q≠0, then this is the case of a stable focal point.
(b) If *p* > 0 and q≠0, then this is the case of an unstable focal point.
(c) If *p* = 0 and q≠0, then $\lambda_{1,2}=p\pm qi$ and there is no net motion toward or away from the equilibrium points, and, in this case, it is the center (or Hopf bifurcation).

**Figure 12:**
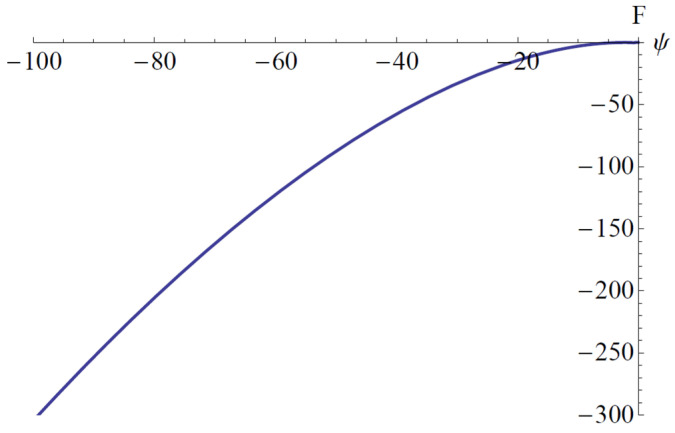
The discriminant F(ψ) given by Equation (94).

**Figure 13:**
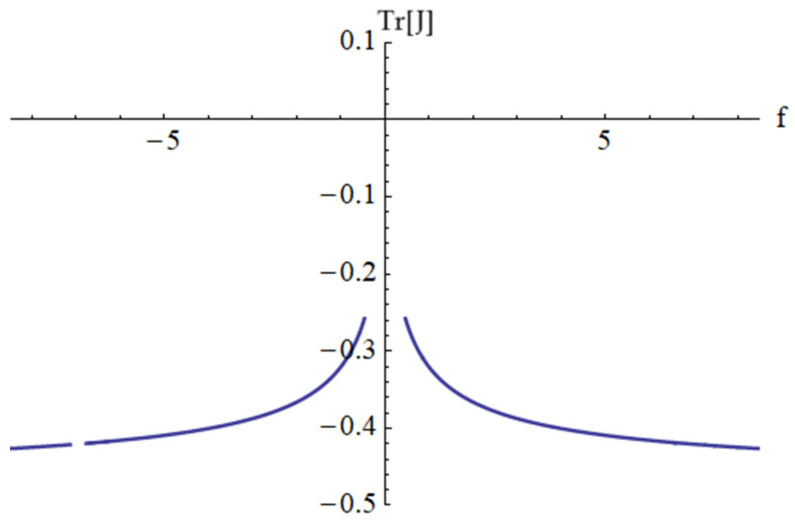
The trace Tr[J] given by Equation (92).

In the particular case of the data presented above, the discriminant F(ψ) given by Equation (94) and the trace Tr(J) given by Equation (92) are depicted in Figure 12 and Figure 13, respectively.

It is clear that we are in case 5.4.a, and accordingly, the equilibrium point is stable.

## 6. Global Stability by the Lyapunov Function

Equations (29) and (30) of the cantilever bimorph piezoelectric beam can be written by adding the control inputs *U*_1_ and *U*_2_ as follows:

(96) $$x_{1}=T_{1},\;{\dot{x}}_{1}={\dot{T}}_{1}=x_{2},\;x_{3}=T_{2},\;{\dot{x}}_{3}={\dot{T}}_{2}$$

(97) $$\begin{array}{c} {\dot{x}}_{2}=-2c_{1}x_{2}\left|x_{2}\right|-\omega^{2}x_{1}-a_{2}x_{1}{\dot{x}}_{2}-a_{3}x_{1}^{3}-a_{4}x_{1}{\dot{x}}_{2}-a_{5}x_{1}^{5}\\ +x_{3}\left(b_{1}+b_{2}x_{1}^{2}+b_{4}x_{1}^{4}\right)+f_{0}sin\bar{\omega }t+U_{1}(x_{1},x_{2},x_{3}) \end{array}$$

(98) $${\dot{x}}_{3}=-\mu x_{3}-x_{2}\left(b_{5}+b_{6}x_{1}^{2}+b_{8}x_{1}^{4}\right)+U_{2}(x_{1},x_{2},x_{3})$$

Defining the tracking errors *E_i_*, *i* = 1, 2, 3 as follows:

(99) $$E_{1}=x_{1}-{\bar{x}}_{1};\;E_{2}=x_{2}-{\dot{\bar{x}}}_{1}+\theta E_{1};\;E_{3}=x_{3}-{\bar{x}}_{3}$$

where ${\bar{x}}_{1}$ and ${\bar{x}}_{3}$ are approximate solutions ${\bar{T}}_{1}$ and ${\bar{T}}_{2}$, respectively, obtained above by OAFM, θ is a positive parameter unknown at this moment, and *U_j_*, *j* = 1, 2 are control inputs.

The estimated parameters are ${\bar{c}}_{1}$, ${\bar{c}}_{2}$, ${\bar{\omega }}^{2}$, ${\bar{a}}_{2}$, ${\bar{a}}_{3}$, ${\bar{a}}_{4}$, ${\bar{a}}_{5}$, $\bar{\mu }$, ${\bar{b}}_{1}$, ${\bar{b}}_{2}$,…, ${\bar{b}}_{7}$ and the estimation error of these parameters is defined as follows [36]:

(100) $$\begin{array}{c} {\tilde{c}}_{1}={\bar{c}}_{1}-c_{1};\;{\tilde{c}}_{2}={\bar{c}}_{2}-c_{2};\;{\tilde{\omega }}^{2}={\bar{\omega }}^{2}-\omega^{2};\;{\tilde{a}}_{i}={\bar{a}}_{i}-a_{i};i=\bar{1,5};\;{\tilde{b}}_{j}=\\ {\bar{b}}_{j}-b_{j},\;j=\bar{1,7};\;\tilde{\mu }=\bar{\mu }-\mu ;\;{\tilde{f}}_{0}={\bar{f}}_{0}-f_{0} \end{array}$$

The Lyapunov function can be defined as follows:

(101) $$\begin{array}{c} V(E_{k},c_{j},\omega^{2},\mu ,U_{i},b_{j})=\frac{1}{2}(\lambda_{1}E_{1}^{2}+\lambda_{2}E_{2}^{2}+\lambda_{3}E_{3}^{2}+2\lambda_{4}{\tilde{c}}_{1}^{2}+\lambda_{5}{\tilde{c}}_{2}^{2}+\lambda_{4}{\tilde{\omega }}^{2}+\\ \lambda_{7}{\tilde{a}}_{2}^{2}+\lambda_{8}{\tilde{a}}_{3}^{2}+\lambda_{9}{\tilde{a}}_{4}^{2}+\lambda_{10}{\tilde{a}}_{5}^{2}+\lambda_{11}{\tilde{b}}_{1}^{2}+\lambda_{12}{\tilde{b}}_{2}^{2}+\lambda_{13}{\tilde{b}}_{4}^{2}+\lambda_{14}{\tilde{b}}_{5}^{2}+\lambda_{15}{\tilde{b}}_{6}^{2}+\\ \lambda_{16}{\tilde{b}}_{7}^{2}+\lambda_{17}{\tilde{\mu }}^{2}) \end{array}$$

where $\lambda_{i},\;i=\bar{1.17}$ are positive parameters. The time derivative of the Lyapunov function can be written taking into account Equations (99)–(101) in the form:

(102) $$\begin{array}{c} \frac{dV}{dt}=\lambda_{1}E_{1}E_{2}-\lambda_{1}\theta E_{1}^{2}+\lambda_{2}E_{2}[2{\tilde{c}}_{1}x_{2}-{\tilde{c}}_{2}x_{2}\left|x_{2}\right|+{\tilde{\omega }}^{2}x_{1}+{\tilde{a}}_{2}{\dot{x}}_{1}x_{2}+{\tilde{a}}_{3}x_{1}^{3}+\\ {\tilde{a}}_{4}x_{1}{\dot{x}}_{2}+{\tilde{a}}_{5}x_{1}^{5}+x_{3}({\tilde{b}}_{1}+{\tilde{b}}_{2}x_{2}^{2}+{\tilde{b}}_{4}x_{1}^{4}+{\tilde{f}}_{0}sin\bar{\omega }t+U_{1}\left(x_{1},x_{2},x_{3}\right)-{\ddot{x}}_{1}+\\ \theta \left(E_{2}-\theta E_{1}\right)]-\lambda_{2}\theta^{2}E_{1}E_{2}+\lambda_{3}E_{3}[\bar{\mu }x_{3}+x_{2}\left({\bar{b}}_{5}+{\bar{b}}_{6}x_{1}^{2}+{\bar{b}}_{7}x_{1}^{2}\right)+\\ U_{2}\left(x_{1},x_{2},x_{3}\right)]+2\lambda_{4}{\tilde{c}}_{1}{\dot{\tilde{c}}}_{1}+\lambda_{5}{\tilde{c}}_{2}{\dot{\tilde{c}}}_{2}+\lambda_{6}\tilde{\omega }\dot{\tilde{\omega }}+\lambda_{7}{\tilde{a}}_{2}{\dot{\tilde{a}}}_{2}+\lambda_{8}{\tilde{a}}_{3}{\dot{\tilde{a}}}_{3}+\lambda_{9}{\tilde{a}}_{4}{\dot{\tilde{a}}}_{4}+\\ \lambda_{10}{\tilde{a}}_{5}{\dot{\tilde{a}}}_{5}+\lambda_{11}{\tilde{b}}_{1}{\dot{\tilde{b}}}_{1}+\lambda_{12}{\tilde{b}}_{2}{\dot{\tilde{b}}}_{2}+\lambda_{13}{\tilde{b}}_{4}{\dot{\tilde{b}}}_{4}+\lambda_{14}{\tilde{b}}_{5}{\dot{\tilde{b}}}_{5}+\lambda_{15}{\tilde{b}}_{6}{\dot{\tilde{b}}}_{6}+\lambda_{16}{\tilde{b}}_{7}{\dot{\tilde{b}}}_{7}\\ +\lambda_{17}\tilde{\mu }\dot{\tilde{\mu }} \end{array}$$

If we define the inputs control *U*_1_ and *U*_2_ through Equation (102) as follows:

(103) $$\begin{array}{c} U_{1}\left(x_{1},x_{2},x_{3}\right)=2{\bar{c}}_{1}x_{2}+{\bar{c}}_{2}x_{2}\left|x_{2}\right|+{\bar{\omega }}^{2}x_{1}+{\bar{a}}_{2}{\dot{x}}_{1}x_{2}+{\bar{a}}_{3}x_{1}^{3}+{\bar{a}}_{4}{x_{1}\dot{x}}_{2}+\\ {\bar{a}}_{5}x_{1}^{5}+x_{3}\left({\bar{b}}_{1}+{\bar{b}}_{2}x_{2}^{2}+{\bar{b}}_{4}x_{1}^{4}-{\bar{f}}_{0}sin\bar{\omega }t\right)+{\ddot{x}}_{1}-\theta E_{1} \end{array}$$

(104) $$U_{2}\left(x_{1},x_{2},x_{3}\right)=\bar{\mu }x_{3}+x_{2}\left({\bar{b}}_{5}+{\bar{b}}_{6}x_{1}^{2}+{\bar{b}}_{7}x_{1}^{4}\right)-{\dot{\bar{x}}}_{3}\;$$

Equation (102) can be rewritten as follows:

(105) $$\begin{array}{c} \frac{dV}{dt}=\left(\lambda_{1}-\theta^{2}\lambda_{2}\right)E_{1}E_{2}-\theta \lambda_{1}E_{1}^{2}+2{\tilde{c}}_{1}\left(\lambda_{2}E_{2}{\bar{x}}_{2}+2\lambda_{4}{\dot{\tilde{c}}}_{1}\right)+{\tilde{c}}_{2}(\lambda_{2}E_{2}{\bar{x}}_{2}\left|{\bar{x}}_{2}\right|+\\ \lambda_{5}{\dot{\tilde{c}}}_{2})+\tilde{\omega }\left(\lambda_{2}E_{2}{\bar{x}}_{1}+\lambda_{6}\dot{\tilde{\omega }}\right)+{\tilde{a}}_{2}\left(\lambda_{2}E_{2}{\bar{x}}_{1}{\ddot{\bar{x}}}_{1}+\lambda_{7}{\dot{\tilde{a}}}_{2}\right)+{\tilde{a}}_{3}\left(\lambda_{2}E_{2}{\bar{x}}_{1}^{3}+\lambda_{8}{\dot{\tilde{a}}}_{3}\right)+\\ {\tilde{a}}_{4}\left(\lambda_{2}E_{2}{\bar{x}}_{1}{\dot{x}}_{1}^{2}+\lambda_{9}{\dot{\tilde{a}}}_{4}\right)+{\tilde{a}}_{5}\left(\lambda_{2}E_{2}{\bar{x}}_{1}^{5}+\lambda_{10}{\dot{\tilde{a}}}_{3}\right)+{\tilde{b}}_{1}\left(\lambda_{2}E_{2}{\bar{x}}_{2}+\lambda_{11}{\dot{\tilde{b}}}_{1}\right)+\\ {\tilde{b}}_{2}\left(\lambda_{2}E_{2}{\bar{x}}_{1}^{2}{\bar{x}}_{2}+\lambda_{12}{\dot{\tilde{b}}}_{2}\right)+{\tilde{b}}_{4}\left(\lambda_{2}E_{2}{\bar{x}}_{1}^{4}{\bar{x}}_{2}+\lambda_{13}{\dot{\tilde{b}}}_{4}\right)+{\tilde{b}}_{5}\left(\lambda_{3}E_{3}{\bar{x}}_{2}+\lambda_{14}{\dot{\tilde{b}}}_{5}\right)+\\ {\tilde{b}}_{6}\left(\lambda_{3}E_{3}{\bar{x}}_{1}^{2}{\bar{x}}_{2}+\lambda_{15}{\dot{\tilde{b}}}_{6}\right)+{\tilde{b}}_{7}\left(\lambda_{3}E_{3}{\bar{x}}_{1}^{4}{\bar{x}}_{2}+\lambda_{16}{\dot{\tilde{b}}}_{7}\right)+\tilde{\mu }\left(\lambda_{3}E_{3}{\bar{x}}_{3}+\lambda_{17}\dot{\tilde{\mu }}\right) \end{array}$$

The estimate parameters in Equation (105): ${\tilde{c}}_{1},{\tilde{c}}_{2},\tilde{\omega },{\tilde{a}}_{i},{\tilde{b}}_{j},{\tilde{f}}_{0},\tilde{\mu }$ are given by the following:

(106) $$\begin{array}{ll} \frac{d{\tilde{c}}_{1}}{dt}=-\frac{1}{2}\frac{\lambda_{2}}{\lambda_{4}}E_{2}x_{2};\; & \frac{d{\tilde{c}}_{2}}{dt}=-\frac{\lambda_{2}}{\lambda_{3}}E_{2}{\bar{x}}_{2}\left|{\bar{x}}_{2}\right|;\;\frac{d\tilde{\omega }}{dt}=-\frac{\lambda_{2}}{\lambda_{6}}E_{2}{\bar{x}}_{1};\;\frac{d{\tilde{a}}_{2}}{dt}\\ & =-\frac{\lambda_{2}}{\lambda_{7}}E_{2}{\bar{x}}_{1}{\dot{\bar{x}}}_{1};\;\frac{d{\tilde{a}}_{3}}{dt}=-\frac{\lambda_{2}}{\lambda_{8}}E_{2}{\bar{x}}_{1}^{3};\;\frac{d{\tilde{a}}_{3}}{dt}=-\frac{\lambda_{2}}{\lambda_{8}}E_{2}{\bar{x}}_{1}^{3};\;\frac{d{\tilde{a}}_{5}}{dt}\\ & =-\frac{\lambda_{2}}{\lambda_{10}}E_{2}{\bar{x}}_{1}^{5};\;\frac{d{\tilde{b}}_{1}}{dt}=-\frac{\lambda_{2}}{\lambda_{11}}E_{2}{\bar{x}}_{2};\;\frac{d{\tilde{b}}_{2}}{dt}\\ & =-\frac{\lambda_{2}}{\lambda_{12}}E_{2}{\bar{x}}_{1}^{2}{\bar{x}}_{2};\;\frac{d{\tilde{b}}_{4}}{dt}=-\frac{\lambda_{2}}{\lambda_{13}}E_{2}{\bar{x}}_{1}^{4}{\bar{x}}_{2};\;\frac{d{\tilde{b}}_{5}}{dt}\\ & =-\frac{\lambda_{3}}{\lambda_{14}}E_{3}x_{2};\;\frac{d{\tilde{b}}_{6}}{dt}=-\frac{\lambda_{3}}{\lambda_{15}}E_{3}x_{1}^{2}x_{2};\;\frac{d{\tilde{b}}_{7}}{dt}\\ & =-\frac{\lambda_{3}}{\lambda_{16}}E_{3}x_{1}^{4}x_{2};\;\frac{d\tilde{\mu }}{dt}=-\frac{\lambda_{3}}{\lambda_{17}}E_{3}x_{3}; \end{array}$$

Equation (105) becomes the following:

(107) $$\frac{dV}{dt}=(\lambda_{1}-\theta^{2}{\lambda_{2})E}_{1}E_{2}-\lambda_{1}\theta E_{1}^{2}$$

The positive parameter θ is defined as follows:

(108) $$\theta =\sqrt{\frac{\lambda_{1}}{\lambda_{2}}}$$

such that Equation (107) can be written in the final form as follows:

(109) $$\frac{dV}{dt}=-\lambda_{1}^{3/2}\lambda_{2}^{-1/2}E_{1}^{2}$$

By employing the Lyapunov function and La Salle’s and Pontryagin’s principles, the system investigated in this study is globally asymptotically stable since the function V is a positively defined function, and dV/dt is a negative definite function.

**Figure 14:**
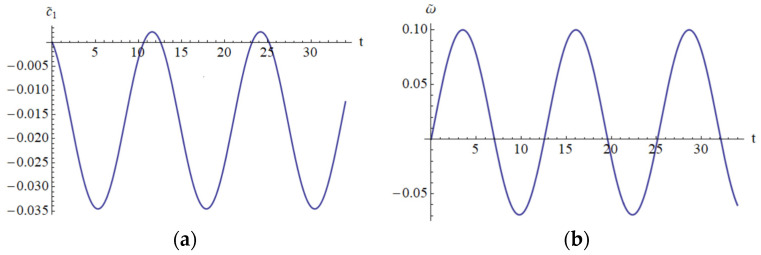
The error on domain [0, 35]: (**a**) The error ${\tilde{c}}_{1}$; (**b**) The error $\bar{\omega }$.

**Figure 15:**
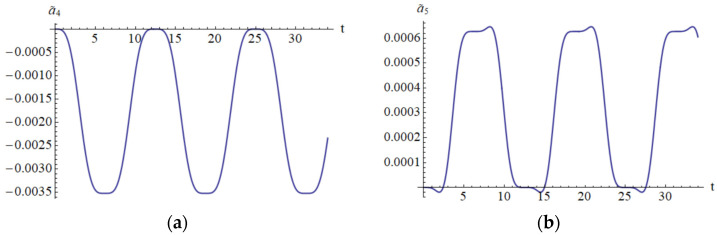
The error on domain [0 to 35]: (**a**) The error ${\bar{a}}_{4}$; (**b**) The error ${\bar{a}}_{5}$.

It is clear that the Lyapunov function depends on the analytical solution obtained by means of OAFM. This is a novel construction that appears for the first time in this paper. In Figure 14 and Figure 15 are depicted the parameters ${\bar{c}}_{1}$, $\bar{\omega }$, ${\bar{a}}_{4}$, ${\bar{a}}_{5}$, which appear in the construction of the Lyapunov function.

## 7. Conclusions

The present study proposed an approximate solution to the nonlinear dynamics of the piezoelectric energy harvesting device subject to mechanical impact and Winkler–Pasternak foundation. Vibration energy harvesters are often linear mass-spring-damper-type devices, but they can have imprecise characteristics of the host environment, and nonlinear behavior could be to improve the response characteristics, such as frequency response. Vibration energy harvesters provide a promising solution to implement self-sustained micro and small electronic devices and sensors. The linear mechanical resonator combined with a piezoelectric transducer is most efficient in the neighborhood of the resonance frequency. The idea of purposefully introducing nonlinearities into the harvester design has been exploited so as to extend its bandwidth and enhance its performance. One class of nonlinear harvesters incorporates cubic, quintic, and quadratic damping nonlinearities.

We considered the simultaneous parametric and external excitation, the strain caused by axial displacement and curvature of the beam, and that the beam is inextensible. The cantilever bimorph piezoelectric energy harvesting beam is subjected to Winkler–Pasternak foundation and mechanical impact. The nonlinear differential governing equations are established by means of extended Hamilton’s principle and discretized by Galerkin–Bubnov. An explicit analytical solution of the frequency response curves is given near the primary resonance. Our procedure is a powerful tool to solve the nonlinear problem with very good accuracy using only the first iteration and only two linear differential equations.

The main novelty of our approach relies on the involvement of a few auxiliary functions, the involvement of the convergence-control parameters, the construction of the initial and the first iteration, and also much freedom to select values of the parameters by rigorous procedures. Unlike other well-known procedures such as the multiple scale method, the Krylov–Bogoliubov method, the Lindstedt–Poincare method, the averaging method, the harmonic balance method, and so on, our technique does not suppose the involvement of a small or large parameter in the governing equation or in the boundary/initial conditions. The auxiliary functions may be determined from the expressions of the nonlinear operator associated with the problem under study. This idea is based on the theory of differential equations related to the Cauchy method, the method of influence functions, the operator method, and others. The presence of these parameters is crucial to assure the accuracy of the solutions and can be found by rigorous mathematical procedures such as the Ritz method, collocation method, least squares method, Galerkin method, Katorovich method, and so on.

The local stability is studied by choosing the equilibrium points and then adopting the Routh–Hurwitz criteria, specifying the existence of a saddle point or Hopf bifurcation. Global stability is performed by the Lyapunov function, Pontryagin, and La Salle’s principle. For the first time, the explicit approximate analytical solution obtained by OAFM is introduced in the construction of the Lyapunov function.

## Data Availability

Data are contained within the article.

## Abbreviations

The following abbreviations are used in this manuscript:

OAFM	Optimal auxiliary functions method
PEH	Piezoelectric energy harvesting

## Appendix A

$$\omega^{2}=\int_{0}^{1}X^{\prime }\left(s\right)X\left(s\right)ds+K_{1}$$

$$\begin{array}{c} a_{2}=\int_{0}^{1}X^{\prime }\left(s\right)X(s)\left(\int_{0}^{s}{X^{\prime }}^{2}ds\right)ds\;-\left(\int_{0}^{1}X”(s)X(s)ds\right)\left[\int_{0}^{1}\left(\int_{0}^{s}{X^{\prime }}^{2}\left(s\right)ds\right)ds\right] \end{array}$$

$$a_{3}=-\frac{3}{2}\int_{0}^{1}X^{IV}\left(s\right){X^{\prime }}^{2}\left(s\right)X\left(s\right)ds-9\int_{0}^{1}X^{\prime \prime \prime }\left(s\right)X”(s)X^{\prime }(s)X(s)ds\;-3\int_{0}^{1}{X”}^{3}\left(s\right)X\left(s\right)ds+K_{3}\int_{0}^{1}X^{4}\left(s\right)d$$

$$a_{4}=\int_{0}^{1}X^{\prime }\left(s\right)X\left(s\right)\left(\int_{0}^{s}{X^{\prime }}^{2}\left(s\right)ds\right)ds\;-\int_{0}^{1}X^{\prime }\left(s\right)X(s)\left[\int_{0}^{1}\left(\int_{0}^{s}{X^{\prime }}^{2}\left(s\right)ds\right)ds\right]ds$$

$$a_{5}=\frac{15}{8}\int_{0}^{1}X^{IV}{X^{\prime }}^{4}\left(s\right)X\left(s\right)ds\;+\frac{45}{2}\int_{0}^{1}{X^{\prime }}^{2}\left(s\right)X”(s)X(s)\left({X”}^{2}\left(s\right)+X^{\prime }\left(s\right)X^{\prime \prime \prime }(s)\right)ds$$

$$b_{1}=X^{\prime }\left(0\right)-X^{\prime }\left(1\right);\;b_{2}=-3\left[X^{\prime }\left(0\right)X(0)-X^{\prime }(1)X”(1)\right];$$

$$b_{4}=\frac{15}{2}\left[{X^{\prime }}^{3}\left(0\right)X(0)-{X^{\prime }}^{3}(1)X(1)\right];b_{5}=\left(\int_{0}^{1}X\left(s\right)ds\right)\left(\int_{0}^{1}X”\left(s\right)ds\right)$$

$$b_{6}=-3\left[\int_{0}^{1}X\left(s\right)ds\right]\left[\int_{0}^{1}{X^{\prime }}^{2}\left(s\right)ds+2\int_{0}^{1}X”(1){X^{\prime }}^{2}(s)ds\right]$$

$$b_{7}=\left[\int_{0}^{1}X\left(s\right)ds\right]\left[\frac{15}{8}\int_{0}^{1}{X^{\prime }}^{4}\left(s\right)ds+\frac{15}{2}\int_{0}^{1}X”(s){X^{\prime }}^{4}(s)ds\right]$$

## Appendix B

(A1)
$$\begin{array}{ll} N\left[T_{10}\right]= & -2c_{1}A\omega sin\omega -\frac{2}{\pi }c_{2}A^{2}\omega^{2}\left(1-\frac{1}{3}cos2\omega t-\frac{1}{15}cos4\omega t-\frac{1}{35}cos6\omega t\ldots \right)\\ & -\frac{1}{2}a_{2}A^{2}\omega^{2}\left(1+cos2\omega t\right)+\frac{1}{4}a_{3}A^{3}\left(cos3\omega t+3cos\omega t\right)\\ & +\frac{1}{4}a_{4}A^{3}\left(cos\omega t-cos3\omega t\right)\\ & +Be^{-\alpha t}cos\Omega t[b_{1}+\frac{1}{4}b_{2}A^{2}\left(1+cos2\omega t\right)+\frac{1}{4}b_{4}A^{4}(3+4cos2\omega t\\ & +cos4\omega t]-f_{0}sin\bar{\omega }t \end{array}$$

(A2)
$$\begin{array}{ll} T_{11}\left(t,\alpha_{i},\beta_{j}\right) & =A_{1}cos\omega t+B_{1}sin\omega t-\frac{\alpha_{1}}{8\omega^{2}}cos3\omega t-\frac{\alpha_{2}}{24\omega^{2}}cos5\omega t\\ & -\frac{\alpha_{3}}{{\bar{\omega }}^{2}-\omega^{2}}cos\bar{\omega }t-\frac{\alpha_{4}}{{9\bar{\omega }}^{2}-\omega^{2}}cos3\bar{\omega }t-\frac{\alpha_{5}}{{25\bar{\omega }}^{2}-\omega^{2}}cos5\bar{\omega }t\\ & -\frac{\beta_{1}}{{\bar{\omega }}^{2}-\omega^{2}}sin\bar{\omega }t-\frac{\beta_{2}}{{9\bar{\omega }}^{2}-\omega^{2}}sin3\bar{\omega }t-\frac{\beta_{3}}{{25\bar{\omega }}^{2}-\omega^{2}}sin5\bar{\omega }t\\ & -\frac{\beta_{4}}{{9\bar{\omega }}^{2}-\omega^{2}}sin3\bar{\omega }t-\frac{\beta_{5}}{{25\bar{\omega }}^{2}-\omega^{2}}sin5\bar{\omega }t \end{array}$$

(A3)
$$\begin{array}{ll} {\bar{T}}_{1}\left(t,C_{1},C_{2},\ldots C_{5}\right) & =Acos\bar{\omega }t+C_{1}(cos\omega t-cos\bar{\omega }t)+C_{2}(3cos\omega t-cos3\bar{\omega }t)\\ & +C_{3}(\bar{\omega }sin\omega t-\omega sin\bar{\omega }t)+C_{4}(\bar{\omega }sin5\omega t-\omega sin5\bar{\omega }t)\\ & +C_{5}(\bar{\omega }sin7\omega t-\omega sin7\bar{\omega }t) \end{array}$$

(A4)
$$\begin{array}{ll} {\bar{T}}_{1}\left(t,C_{1},C_{2},\ldots C_{4}\right)= & Acos\omega t+C_{1}(cos\omega t-cos\bar{\omega }t)+C_{2}(cos3\omega t-cos3\bar{\omega }t)\\ & +C_{3}(\bar{\omega }sin\omega t-\omega sin\bar{\omega }t)+C_{4}(\bar{\omega }sin5\omega t-\omega sin5\bar{\omega }t) \end{array}$$

(A5)
$$\begin{array}{ll} \omega^{2}\left(\frac{\partial^{2}T_{10}}{\partial \xi_{1}^{2}}+p\frac{\partial^{2}T_{11}}{\partial \xi_{1}^{2}}\right) & +2\omega p\left(\frac{\partial^{2}T_{10}}{\partial \xi_{1}\partial \eta }+p\frac{\partial^{2}T_{11}}{\partial \xi_{1}\partial \eta }\right)+p^{2}\left(\frac{\partial^{2}T_{10}}{\partial \eta^{2}}+p\frac{\partial^{2}T_{11}}{\partial \eta^{2}}\right)+\omega^{2}\left(T_{10}+pT_{11}\right)\\ & +p\left\{2c_{1}\left[\omega \left(\frac{\partial T_{10}}{\partial \xi_{1}}+p\frac{\partial T_{11}}{\partial \xi_{1}}\right)+p\left(\frac{\partial T_{10}}{\partial \eta }+p\frac{\partial T_{11}}{\partial \eta }\right)\right]\right.\\ & +c_{2}\left[\omega \left(\frac{\partial T_{10}}{\partial \xi_{1}}+p\frac{\partial T_{11}}{\partial \xi_{1}}\right)+p\left(\frac{\partial T_{10}}{\partial \eta }+p\frac{\partial T_{11}}{\partial \eta }\right)\right]\left|\omega \left(\frac{\partial T_{10}}{\partial \xi_{1}}+p\frac{\partial T_{11}}{\partial \xi_{1}}\right)+p\left(\frac{\partial T_{10}}{\partial \eta }+p\frac{\partial T_{11}}{\partial \eta }\right)\right|\\ & +a_{2}[\omega^{2}(T_{10}\frac{\partial^{2}T_{10}}{\partial \eta^{2}}+p\left(T_{10}\frac{\partial^{2}T_{11}}{\partial \eta^{2}}+T_{11}\frac{\partial^{2}T_{11}}{\partial \eta^{2}}\right)+p^{2}T_{11}\frac{\partial^{2}T_{11}}{\partial \xi_{1}^{2}})\\ & +2\omega p\left(T_{10}\frac{\partial^{2}T_{10}}{\partial \xi_{1}\partial \eta }+p(T_{10}\frac{\partial^{2}T_{11}}{\partial \xi_{1}\partial \eta }+T_{11}\frac{\partial^{2}T_{10}}{\partial \xi_{1}\partial \eta }\right)+p^{2}T_{11}\frac{\partial^{2}T_{11}}{\partial \xi_{1}\partial \eta })\\ & +p^{2}\left(T_{10}\frac{\partial^{2}T_{10}}{\partial \eta^{2}}+p\left(T_{11}\frac{\partial^{2}T_{10}}{\partial \eta^{2}}+T_{10}\frac{\partial^{2}T_{11}}{\partial \eta^{2}}\right)+p^{2}T_{11}\frac{\partial^{2}T_{11}}{\partial \eta^{2}}\right)]\\ & +a_{3}\left(T_{10}^{3}+3pT_{10}^{2}T_{11}+3p^{2}T_{10}T_{11}^{2}+p^{3}T_{11}^{3}\right)\\ & +a_{4}[\omega^{2}(T_{10}{\left(\frac{\partial T_{10}}{\partial \xi_{1}}\right)}^{2}+p\left(T_{11}{\left(\frac{\partial T_{10}}{\partial \xi_{1}}\right)}^{2}+2T_{10}\frac{\partial T_{10}}{\partial \xi_{1}}\frac{\partial T_{11}}{\partial \xi_{1}}\right)+p^{2}\left(2T_{11}\frac{\partial T_{10}}{\partial \xi_{1}}\frac{\partial T_{11}}{\partial \xi_{1}}+T_{10}{\left(\frac{\partial T_{11}}{\partial \xi_{1}}\right)}^{2}\right)\\ & +p^{3}T_{11}{\left(\frac{\partial T_{11}}{\partial \xi_{1}}\right)}^{2})\\ & +2\omega p(T_{10}\frac{\partial T_{10}}{\partial \xi_{1}}\frac{\partial T_{11}}{\partial \eta }\\ & +p(T_{11}\frac{\partial T_{10}}{\partial \xi_{1}}\frac{\partial T_{10}}{\partial \eta }+p\left(T_{11}\frac{\partial T_{10}}{\partial \xi_{1}}\frac{\partial T_{11}}{\partial \eta }+T_{10}\frac{\partial T_{11}}{\partial \xi_{1}}\frac{\partial T_{10}}{\partial \eta }+T_{10}\frac{\partial T_{10}}{\partial \xi_{1}}\frac{\partial T_{11}}{\partial \eta }\right)\\ & +p^{2}\left(T_{11}\frac{\partial T_{11}}{\partial \xi_{1}}\frac{\partial T_{10}}{\partial \eta }+T_{11}\frac{\partial T_{10}}{\partial \xi_{1}}\frac{\partial T_{11}}{\partial \eta }\right))\\ & +p^{2}(T_{10}{\left(\frac{\partial T_{10}}{\partial \eta }\right)}^{2}+p\left(T_{11}{\left(\frac{\partial T_{10}}{\partial \eta }\right)}^{2}+2T_{10}\frac{\partial T_{10}}{\partial \eta }\frac{\partial T_{11}}{\partial \eta }\right)+p^{2}\left(2T_{11}\frac{\partial T_{10}}{\partial \eta }\frac{\partial T_{11}}{\partial \eta }+T_{10}{\left(\frac{\partial T_{11}}{\partial \eta }\right)}^{2}\right)\\ & +p^{3}T_{11}{\left(\frac{\partial T_{11}}{\partial \eta }\right)}^{2}))]+a_{5}\left(T_{10}^{5}+5pT_{10}^{4}T_{11}+10T_{10}^{3}T_{11}^{2}+10p^{3}T_{10}^{2}T_{11}^{3}+5p^{4}T_{10}T_{11}^{4}+p^{5}T_{11}^{5}\right)\\ & +b_{1}\left(T_{20}+pT_{21}\right)+b_{2}\left[T_{20}T_{10}^{2}+p\left(T_{21}T_{10}^{2}+2T_{20}T_{10}T_{11}\right)+p^{2}\left(2T_{21}T_{10}T_{11}+T_{20}T_{11}^{2}\right)+p^{3}T_{21}T_{11}^{2}\right]\\ & +b_{4}[T_{20}T_{10}^{4}+p\left(T_{21}T_{10}^{4}+4T_{20}T_{10}^{3}T_{11}\right)+p^{2}\left(4T_{21}T_{10}^{3}T_{11}+6T_{20}T_{10}^{2}T_{11}^{2}\right)+p^{3}\left(6T_{21}T_{10}^{2}T_{11}^{2}+4T_{20}T_{10}T_{11}^{3}\right)\\ & +p^{4}\left(T_{20}T_{11}^{4}+4T_{21}T_{10}T_{11}^{3}\right)]-f_{0}sin\omega t\}=0 \end{array}$$

(A6)
$$\begin{array}{ll} \Omega \left(\frac{\partial T_{20}}{\partial \xi_{2}}+p\frac{\partial T_{21}}{\partial \xi_{2}}\right) & +p\left(\frac{\partial T_{20}}{\partial \eta }+p\frac{\partial T_{21}}{\partial \eta }\right)+\Omega \left(T_{20}+pT_{21}\right)\\ & +p\left\{\left(\mu -\Omega \right)\left(T_{20}+pT_{21}\right)+b_{5}\left[\omega \left(\frac{\partial T_{10}}{\partial \xi_{1}}+p\frac{\partial T_{21}}{\partial \xi_{1}}\right)+p\left(\frac{\partial T_{10}}{\partial \eta }+p\frac{\partial T_{11}}{\partial \eta }\right)\right]\right.\\ & +b_{6}[\omega T_{10}^{2}\frac{\partial T_{11}}{\partial \xi_{1}}+p\left(2\omega T_{10}T_{11}\frac{\partial T_{10}}{\partial \xi_{1}}+T_{10}^{2}\left(\frac{\partial T_{11}}{\partial \xi_{1}}+\frac{\partial T_{10}}{\partial \eta }\right)\right)\\ & +p^{2}\left(2T_{10}T_{11}\frac{\partial T_{10}}{\partial \xi_{1}}+2\omega \frac{\partial T_{11}}{\partial \xi_{1}}T_{10}T_{11}+\omega T_{11}^{2}\frac{\partial T_{10}}{\partial \xi_{1}}\right)+p^{3}\left(\omega T_{11}^{2}\frac{\partial T_{11}}{\partial \xi_{1}}+T_{11}^{2}\frac{\partial T_{10}}{\partial \eta }\right)+p^{4}T_{11}^{2}\frac{\partial T_{10}}{\partial \eta }]\\ & +b_{7}[\omega T_{10}^{4}\frac{\partial T_{10}}{\partial \xi_{1}}+p\left(4\omega T_{10}^{3}T_{11}\frac{\partial T_{10}}{\partial \xi_{1}}+T_{10}^{4}\frac{\partial T_{11}}{\partial \eta }\right)+p^{2}\left(4\omega T_{10}^{3}\frac{\partial T_{11}}{\partial \xi_{1}}+T_{10}^{4}\frac{\partial T_{11}}{\partial \eta }+6\omega T_{10}^{2}T_{11}^{2}\frac{\partial T_{10}}{\partial \xi_{1}}\right)\\ & +p^{3}\left(4\omega T_{10}T_{11}^{3}+6\omega \frac{\partial T_{11}}{\partial \xi_{1}}T_{10}^{2}T_{11}^{2}+6T_{10}^{2}T_{11}^{2}\frac{\partial T_{11}}{\partial \eta }\right)+p^{4}\left({\omega T}_{11}^{4}\frac{\partial T_{10}}{\partial \xi_{1}}+4\omega T_{10}T_{21}^{3}\frac{\partial T_{11}}{\partial \xi_{1}}+4T_{10}T_{11}^{3}\frac{\partial T_{10}}{\partial \eta }\right)\\ & +p^{5}T_{11}^{4}\left(\omega \frac{\partial T_{11}}{\partial \xi_{1}}+\frac{\partial T_{11}}{\partial \eta }\right)+p^{6}T_{11}^{4}\frac{\partial T_{11}}{\partial \eta }]\}=0 \end{array}$$
